# Investigating Heterogeneity in Response Strategies: A Mixture Multidimensional IRTree Approach

**DOI:** 10.1177/00131644231206765

**Published:** 2023-11-09

**Authors:** Ö. Emre C. Alagöz, Thorsten Meiser

**Affiliations:** 1 University of Mannheim, Germany

**Keywords:** response styles, item response theory, mixture models, IRTree, response strategies

## Abstract

To improve the validity of self-report measures, researchers should control for response style (RS) effects, which can be achieved with IRTree models. A traditional IRTree model considers a response as a combination of distinct decision-making processes, where the substantive trait affects the decision on response direction, while decisions about choosing the middle category or extreme categories are largely determined by midpoint RS (MRS) and extreme RS (ERS). One limitation of traditional IRTree models is the assumption that all respondents utilize the same set of RS in their response strategies, whereas it can be assumed that the nature and the strength of RS effects can differ between individuals. To address this limitation, we propose a mixture multidimensional IRTree (MM-IRTree) model that detects heterogeneity in response strategies. The MM-IRTree model comprises four latent classes of respondents, each associated with a different set of RS traits in addition to the substantive trait. More specifically, the class-specific response strategies involve (1) only ERS in the “ERS only” class, (2) only MRS in the “MRS only” class, (3) both ERS and MRS in the “2RS” class, and (4) neither ERS nor MRS in the “0RS” class. In a simulation study, we showed that the MM-IRTree model performed well in recovering model parameters and class memberships, whereas the traditional IRTree approach showed poor performance if the population includes a mixture of response strategies. In an application to empirical data, the MM-IRTree model revealed distinct classes with noticeable class sizes, suggesting that respondents indeed utilize different response strategies.

## Introduction

Questionnaires are common tools in psychological and educational research to measure unobservable constructs (e.g., personality traits; Caprara et al., 1993) in large samples efficiently and inexpensively. However, many factors can damage the quality of questionnaire data, such as item wording effects (Nye et al., 2010), item position effects (Gehlbach & Barge, 2012; Tourangeau & Rasinski, 1988), socially desirable responding (Ones et al., 1996), and response styles (RS). RS are defined as tendencies to choose specific response categories regardless of the item content (Baumgartner & Steenkamp, 2001; Paulhus, 1991). Two commonly reported kinds of RS are extreme RS (ERS) and midpoint RS (MRS). ERS is defined as the tendency to choose the extreme response categories, whereas MRS is defined as the tendency to choose the middle response category.

Respondents can make use of RS as a way of heuristic responding, thereby they aim to reduce the cognitive and time cost of responding to questionnaire items (Krosnick, 1991; Krosnick et al., 1996; Podsakoff et al., 2012). If not controlled for, RS can bias trait estimates (Bolt & Johnson, 2009), score correlations and structural relationships between traits (Böckenholt & Meiser, 2017; Tutz et al., 2018), and psychometric properties of the questionnaire (Ashton et al., 2017; Kreitchmann et al., 2019; Plieninger, 2017). Moreover, there is a substantial amount of research that found between-group differences in RS as a source for measurement non-invariance (D’Urso et al., 2022; Eid & Rauber, 2000; Liu et al., 2017).

Item Response Theory (IRT) models are popular tools for dealing with RS. IRT-based approaches model RS either with person-specific threshold shifts (Falk & Cai, 2016; Jin & Wang, 2014) or additional traits influencing respondents’ category choices, for instance, in the multidimensional nominal response model (MNRM; Bolt & Johnson, 2009; for an overview, see Henninger & Meiser, 2020a). If researchers opt for the additional traits approach, they can either model a priori specified RS with fixed scoring weights (e.g., a trait that makes respondents favor the extreme categories) or estimate these scoring weights and interpret the meaning of RS traits post hoc.

A more recent approach, item response tree (IRTree) models, can also be used to control for a priori specified RS (Böckenholt & Meiser, 2017). IRTrees capture the cognitive processes underlying responding by modeling an observed response as a product of several decision-making processes. There are many different uses of IRTree models depending on the theoretical assumptions and the number of response categories (for an overview, see Böckenholt, 2012; De Boeck & Partchev, 2012; Jeon & De Boeck, 2016). When the aim is to separate the substantive trait from RS traits, researchers usually model the following three decisions for a 5-point rating scale item: (1) whether to choose the middle category (i.e., judgment), (2) whether to agree with the item content (i.e., direction), and (3) whether to choose an extreme category (i.e., intensity). RS are defined a priori in a way that the outcomes of the judgment, direction, and intensity decisions are determined by the MRS, the substantive, and the ERS traits, respectively. Therefore, only the response direction is determined by the substantive trait, whereas choosing a middle or extreme category is largely determined by RS traits.

Regardless of an exploratory approach or a priori specification of RS, the models being used imply that all respondents use the same set of RS while responding. For example, the MNRM assumes that the same RS traits and their scoring weights influence the category choices of all respondents in the same way, and IRTree models assume that MRS and ERS traits influence the judgment and extremity decisions of all respondents. However, respondents might differ in their response strategies, such that some respondents might use only the substantive trait, some might use only one type of RS, and some might use all possible RS while responding to an item. These between-person differences in response strategies cause between-person differences in measurement models and, thus, measurement non-invariance. Ignoring measurement non-invariance and fitting only one measurement model for all respondents is likely to yield biased estimates and threaten the validity of our inferences about persons, groups, and test characteristics (Vandenberg & Lance, 2000; Wicherts, 2016).

There can be several reasons for differential use of RS among respondents. First, respondents with better cognitive skills, stronger motivation, or better knowledge about the research topic may spend their resources on deciding on the response option that describes them best. Therefore, their responses will be less affected by RS and will mainly determined by the substantive trait instead. Indeed, Khorramdel and colleagues (2019) found that respondents with higher cognitive literacy and numeracy scores used ERS to a lesser extent. Second, if respondents lack the motivation to participate in a study or lack related cognitive skills, they can incorporate RS in their response strategies to minimize their cognitive efforts (i.e., heuristic responding, Galesic et al., 2008; Krosnick, 1991). However, it does not necessarily mean that respondents use all types of RS at once or randomly choose one type of RS and make use of it. There can be situational (Shulman, 1973), demographic (Krosnick et al., 1996; Meisenberg & Williams, 2008), or individual covariates (e.g., personality traits) that determine which specific RS are employed by respondents to achieve effort minimization.

## Approaches to Detect Heterogeneity in Response Strategies

If there are latent subpopulations of respondents following different response strategies, we do not know which respondents belong to which subpopulation in advance. In other words, there is not an observed grouping variable that indicates which response strategy a respondent uses. For such problems, the most suitable approach is to make use of mixture models (Lindsay, 1995; McLachlan & Basford, 1988). Mixture models assume that the data contains multiple subpopulations of respondents (i.e., latent classes), each of which follows a different type of distribution or a common type of distribution with different parameter values.

For modeling heterogeneity in response behaviors, earlier studies used mixture Rasch models (Rost, 1990; Wetzel et al., 2016), mixture (generalized) partial credit models (gPCM, Huang, 2016; Rost, 1991), HYBRID models (von Davier & Yamamoto, 2004; Yamamoto, 1982; Yamamoto & Everson, 1995), and mixture IRTree models (Khorramdel et al., 2019; Kim & Bolt, 2021; Tijmstra et al., 2018). Mixture Rasch and PCM models capture subpopulations of respondents who differ in threshold parameters. Therefore, these models detect respondents who merely differ in what part of the response scale they tend to use (Henninger & Meiser, 2020a). However, we are interested in detecting respondents who differ in their response strategies that stem from different underlying cognitive processes. Therefore, mixture Rasch and PCM models are not considered in this study. Two other model families, HYBRID and confirmatory mixture IRTrees, handle responding as a cognitive process and investigate heterogeneity in these processes with some assumptions about the response strategies used in their mixture components.

### HYBRID Model

The HYBRID model proposed by Yamamoto (1982) is specified to have two classes: an IRT class and an independence class. In the first class, an IRT model holds. In the second class, an independence model holds that corresponds to traditional latent class analysis (von Davier & Rost, 2006; von Davier & Yamamoto, 2004, 2007). Responses are determined by a substantive trait in the IRT class, whereas respondents randomly choose their responses in the independence class. Although this line of research accounts for heterogeneity in cognitive processes underlying response behavior, it still assumes homogeneity of response strategies in the IRT class.

### General Mixture Item Response Models

Tijmstra and colleagues (2018) proposed a confirmatory mixture item response model for 5-point rating items to identify respondents who use the middle category to reflect a nonresponse choice (IRTree class) and those who use it to indicate their endorsement level (gPCM; generalized partial credit model class). In the IRTree class, respondents first make a binary decision on whether to choose the middle category, which is affected by an MRS trait. If the middle category is not chosen, then they decide which of the other four reflects their endorsement level, which is determined by the substantive trait through a gPCM in compliance with categories’ ordinal interpretation. In the gPCM class, the middle category is also interpreted as part of the ordinal scale of item endorsement. Therefore, responses in this class are modeled with a unidimensional gPCM, where a substantive trait affects the probability of all five categories. Tijmstra and colleagues (2018) found that around 30% of the respondents interpret the middle category as a nonresponse, whereas the rest consider it as part of the ordinal scale.

There are some drawbacks to the mixture model of Tijmstra and colleagues (2018). In their model, an extreme response can occur only because of a high or low substantive trait score, as extreme categories are part of a unidimensional gPCM in both classes. However, earlier studies reported that ERS can have a significant impact on choosing extreme categories. To account for that, one can extend their model to accommodate an ERS trait in gPCM parts. Nevertheless, even if ERS is incorporated in their mode, there can be heterogeneity in the source of extreme category choices just like for the middle category choices, and one must further account for it.

In this study, we propose a new mixture model that is fully in the IRTree framework, detects heterogeneity in the use of middle category and extreme categories, and can be extended to any number of categories.

### Stepwise Mixture IRTree Approach

Khorramdel and colleagues (2019) proposed a stepwise mixture multidimensional IRTree model to differentiate respondents who use only the substantive trait and those who use the substantive trait and ERS trait. It is a three-step approach involving the following steps: (1) testing whether RS exists in the data via IRTree models, (2) testing whether heterogeneity in RS exists via a mixture 2PL model on IRTree data, (3) fitting IRTree models in each class to see if and which RS exist in that class. With this approach, the first step can yield erroneous results if there is heterogeneity in RS use that is yet to be detected in the second step; therefore, the first step can be skipped, as the authors also suggest. Furthermore, their approach rules out an effect of the substantive trait on response intensity, while an extreme response can actually indicate an extreme opinion.

### A Mixture IRTree Model for Extreme RS

**Figure 1.**
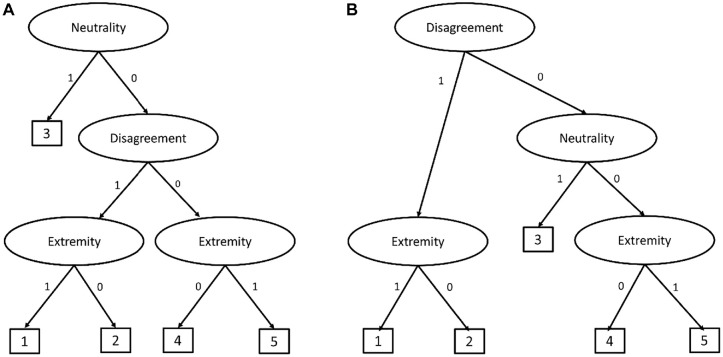
(A) Tree Structure in Böckenholt (2012). (B) Tree Structure of M-IRTree.

Recently, Kim and Bolt (2021) proposed a 2-class confirmatory mixture IRTree model that can distinguish respondents, who use the substantive trait for the response direction and ERS for the response intensity, from those who use the substantive trait for both the response direction and intensity. Their model is well suited for detecting heterogeneity in the use of ERS with 4-point rating items. However, when we have five or a higher number of categories, then we also need to model the MRS effects and heterogeneity in the use of MRS. For 5-point rating items, we can extend their model to have an additional decision node for the middle category choices affected by an MRS factor (see Figure 1 and Böckenholt & Meiser, 2017). Then, it becomes cumbersome to model the heterogeneity in the use of MRS because this traditional IRTree structure does not include the middle category in the ordinal response process. In other words, we cannot specify an alternative class where the middle category is chosen due to moderate level of substantive trait (see the next section for further details). To simultaneously model heterogeneity in the sources of middle and extreme category choices, a different IRTree structure that does not separate the middle category from the response process is needed. The present study proposes a new IRTree structure for 5-point rating items, including MRS effects, incorporating the middle category into the ordinal endorsement scale, and modeling the heterogeneity in the sources of both extreme and middle categories simultaneously.

In the present study, we propose a confirmatory mixture multidimensional IRTree (MM-IRTree) model. The MM-IRTree model detects respondent subpopulations that differ in what kinds of RS they use in their response strategies in addition to the substantive trait that is to be measured. More specifically, the proposed MM-IRTree model consists of four classes that are defined as the following: (1) respondents who use only ERS, (2) respondents who use only MRS, (3) respondents who use both ERS and MRS, (4) respondents who use neither ERS nor MRS. The MM-IRTree framework offers at least three advantages to researchers. First, it functions on 5-point rating items that are commonly used in psychological and educational measurement. Second, it accounts for the heterogeneity in the sources of middle-category and extreme-category responses simultaneously. Third, it purifies the substantive trait estimates from the effects of stylistic responding by acknowledging that each respondent can be contaminated by a different set of RS.

The next section introduces the new multidimensional IRTree model for 5-point rating items and its mixture extension. Then, we describe the model estimation, simulation settings, and simulation results. Next, we illustrate the proposed model by analyzing an empirical data set. We end the paper with a conclusion and discussion.

## IRTree Models

We present an example IRTree model for 5-point rating items from Böckenholt (2012) in Figure 1(A). In the example IRTree model, respondents make up to three decisions until they reach a response. At the first node, they decide whether they give a judgment about the item content or pick a neutral middle category. If they give a judgment, then, they decide whether to agree or disagree with the item content at the second node. Finally, they decide whether to choose an extreme or intermediate category at the third node. By relating the MRS trait to the first, the substantive trait to the second, and the ERS trait to the third nodes, the effects of RS on the measurement of the substantive trait are accommodated (for further technical details, we refer readers to Böckenholt, 2012; Böckenholt & Meiser, 2017).

There are two limitations to this example IRTree model. First, the middle category is treated as a non-informative neutral response and separated from the response process. However, respondents can choose the middle category not only because they have a high MRS score but also because of a medium substantive trait value (Tijmstra et al., 2018). Second, the IRTree model in Figure 1(A) assumes that the substantive trait only affects the binary decision between agreement and disagreement. This assumption disregards the possible information about the substantive trait that would come from the middle and extreme categories. Indeed, previous research showed that the substantive trait also plays a role in the fine-grained selection among relative disagreement and agreement categories for 6-point rating items (Meiser et al., 2019). Therefore, we introduce a multidimensional nodes IRTree (M-IRTree) model for 5-point rating items that allow the substantive trait to affect middle category choices and fine-grained selection among the agreement and disagreement categories.

### Multidimensional Nodes IRTree Model

As a first step to introduce the M-IRTree model for 5-point rating items, we describe the decisions involved in the response process. According to the M-IRTree, respondents make up to three judgments, as depicted in Figure 1(B). At the first node, respondents decide whether to disagree with the item content or not. If the decision is disagreement, respondents go to the extremity node to decide whether they choose the extreme disagreement category. If the decision is against disagreement, respondents proceed to the neutrality node to decide whether they stay neutral or agree with the item content. If they decide to stay neutral, they choose the middle category. If they decide to agree, in contrast, they go to the extremity node, where they decide whether to choose the extreme agreement category. It is important to note that the first two nodes are actually a sequential IRT model, where disagreement, neutrality, and agreement are modeled as ordinal outcomes (Tutz, 1990). Therefore, we addressed the first limitation of the IRTree model in Figure 1(A) by allowing the substantive trait to play a role at the neutrality node.^1^

The second step of model specification is to decompose items into pseudo-items. According to the M-IRTree structure presented in Figure 1(B), an observed rating response is decomposed into three pseudo-items that represent the disagreement node (i.e., $Y_{0}^{*})$ , the neutrality node (i.e., $Y_{1}^{*}$ ), and the extremity node (i.e., $Y_{2}^{*}$ ). In the left-hand side of Table 1, we present these pseudo-items and which values they are assigned for a given original response category. For instance, to give a response of “3,” respondents do not endorse the disagreement node but only endorse the neutrality node. So, the first pseudo-item is coded “0,” the second pseudo-item is coded “1,” and the third pseudo-item is missing by design since the extremity node is not involved.

**Table 1.** Pseudo-Items, Value Assignments for Original Response Categories, and Response Probabilities of Each Pseudo-Item.

Pseudo-items	Original Response Categories					$\mathrm{Pr}(Y_{\mathrm{pkj}}^{*}=y_{\;\mathrm{pkj}}^{*})$
	1	2	3	4	5	
$y_{\;p0j}^{*}$ (Disagreement)	1	1	0	0	0	$g^{-1}\left(y_{\;p0j}^{*}\left[-\alpha_{j}^{\;\theta }\theta_{p}-\alpha_{j}^{\;\mathrm{mrs}}\eta_{p}^{\;\mathrm{mrs}}-\beta_{0j}\right]\right)$
$y_{\;p1j}^{*}$ (Neutrality)	-	-	1	0	0	$g^{-1}\left(y_{\;p1j}^{*}\left[-\alpha_{j}^{\;\theta }\theta_{p}+\alpha_{j}^{\mathrm{mrs}}\eta_{p}^{\;\mathrm{mrs}}-\beta_{1j}\right]\right)$
$y_{\;p2j}^{*}$ (Extremity)	1	0	-	0	1	$g^{-1}\left(y_{\;p2j}^{*}\left[\pm \omega \alpha_{j}^{\;\theta }\theta_{p}+\;\alpha_{j}^{\;\mathrm{ers}}\eta_{p}^{\;\mathrm{ers}}-\beta_{2j}\right]\right)$

*Note. A cell with*“-” represents missing by design. The sign of the factor loadings of θ at the extremity node changes depending on the outcome of the disagreement pseudo-item.

The third step is to relate latent variables to these three nodes with a 2PL item response model. The decision about which latent variables should be involved at which nodes depends on what we aim to model. Assume that we aim to model both ERS and MRS while also allowing the substantive trait to affect the middle and extreme categories. Then, we can relate the substantive trait, ERS trait, and MRS trait to the M-IRTree nodes as follows:

- **Disagreement Node:** Higher values of the substantive trait and of the MRS trait increase the probability of response categories “3,”“4,” and “5,” and decrease the probability of disagreement categories “1” and ”2.” Therefore, the substantive trait and the MRS trait have negative factor loadings at the first node.
- **Neutrality Node:** Higher values of the substantive trait increase the probability of agreement categories “4” and “5” and decrease the probability of middle category “3.” In contrast, higher values of the MRS trait increase the probability of the middle category “3” and decrease the probability of agreement categories “4” and “5.” Therefore, the substantive trait has negative factor loadings, and the MRS has positive factor loadings at the second node. Here, at the end of the first two nodes, we can see that higher MRS values increase the probability of the middle category and decrease the probability of both the disagreement and agreement categories. Meanwhile, higher substantive trait values consistently favor the higher categories.
- **Extremity Nodes:** Higher values of the substantive trait increase the probability of extreme agreement category “5” but decrease the probability of extreme disagreement category “1,” while higher values of the ERS trait increase the probability of both extreme agreement and disagreement categories “5” and “1.” Therefore, the substantive trait has negative factor loadings at the disagreement side and positive factor loadings at the agreement side of the third node. The ERS trait has positive factor loadings at both sides of the third node. At this node, we address the second limitation of the IRTree model in Figure 1(A) by allowing the substantive trait to affect extremity decisions.

The fourth and the last step is to formally express the item response model for the original response categories. Let $Y_{\mathrm{pj}}\in \left\{1,2,3,4,5\right\}$ denote the original response category for subject *p* and item *j*. Then, let $\theta_{p}$ , $\eta_{p}^{\;\mathrm{ers}}$ , and $\eta_{p}^{\;\mathrm{mrs}}$ denote the values of the substantive trait, the ERS trait, and the MRS trait for subject *p*. Next, let $\alpha_{j}^{\;\theta }$ , $\alpha_{j}^{\;\mathrm{ers}}$ , and $\alpha_{j}^{\;\mathrm{mrs}}$ denote factor loadings of the substantive trait, the ERS trait, and the MRS trait for item *j*. Finally, let ω be a proportionality constant of the substantive trait at the extremity node.^2^ Then, the ω parameter implies that the substantive trait is involved in the extremity nodes, but its effect is proportional to its effect on the first two nodes. Naturally, ω<1(ω>1) means that the substantive trait has a smaller (larger) effect at the third node than at the first two nodes. Next, $\beta_{0j}$ , $\beta_{1j}$ , and $\beta_{2j}$ are the difficulty-related parameters at the first, second, and third nodes. Note that we simply refer to them as difficulty parameters, but they are not interpreted as in the unidimensional IRT models.^3^ Finally, $g^{-1}$ denotes a logistic function but can be replaced with a probit if preferred (e.g., Böckenholt, 2019). On the right-hand side of Table 1, we provide the node-specific response probabilities. Since the probability of observing an original response category is the product of node probabilities leading to that response in the tree structure, the item response model defining the probabilities of the original response categories is:

(1) $$\begin{array}{c} \mathrm{Pr}\left(y_{\mathrm{pj}}|\theta_{p},\eta_{p}^{\;\mathrm{ers}},\eta_{p}^{\;\mathrm{mrs}},\alpha_{j}^{\;\theta },\alpha_{j}^{\;\mathrm{ers}},\alpha_{j}^{\;\mathrm{mrs}},\omega ,\beta_{0j},\beta_{1j},\beta_{2j}\right)\\ =g^{-1}\left(y_{\;p0j}^{*}\left[-\alpha_{j}^{\;\theta }\theta_{p}-\alpha_{j}^{\;\mathrm{mrs}}\eta_{p}^{\;\mathrm{mrs}}-\beta_{0j}\right]\right)\\ \times g^{-1}{\left(y_{\;p1j}^{*}\left[-\alpha_{j}^{\;\theta }\theta_{p}+\alpha_{j}^{\;\mathrm{mrs}}\eta_{p}^{\;\mathrm{mrs}}-\beta_{1j}\right]\right)}^{I\left(y_{\mathrm{pj}}\geq 3\right)}\\ \times g^{-1}{\left(y_{\;p2j}^{*}\left[\pm \alpha_{j}^{\;\theta }\omega \theta_{p}+\alpha_{j}^{\;\mathrm{ers}}\eta_{p}^{\;\mathrm{ers}}-\beta_{2j}\right]\right)}^{I\left(y_{\mathrm{pj}}\neq 3\right)} \end{array}$$

The M-IRTree model assumes that all respondents make use of both ERS and MRS traits in their response strategy. However, if there are subpopulations of respondents who use only ERS, only MRS, or neither of them, M-IRTree would yield biased estimates as it assumes all respondents make use of both types of RS to the same extent.

### Mixture Multidimensional Nodes IRTree Model

The proposed MM-IRTree model is a confirmatory mixture model with four latent classes. In all classes, the substantive trait is involved in the disagreement, neutrality, and extremity nodes in the way that the higher the substantive trait score, the higher the probability of choosing higher categories (see Table 1). However, the four classes differ in what RS respondents use as part of their response strategy. In the “ERS only” class, respondents use the ERS trait at the extremity nodes. In the “MRS only” class, respondents use the MRS trait at the disagreement and neutrality nodes. In the “2RS” class, respondents use the ERS trait at the extremity node and the MRS trait at the disagreement and neutrality nodes, while respondents use neither the ERS trait nor MRS trait as part of their response strategy in the “0RS” class.

We transcribe these four class definitions into our model by fixing the factor loadings of ERS and MRS traits at zero when they are not used in the response strategy of a class. Formally, the following factor loadings are fixed to zero: (1) MRS factor loadings in the “ERS only” class, (2) ERS factor loadings in the “MRS only” class, (3) both ERS and MRS factor loadings in the “0RS” class. Factor loadings of ERS and MRS traits in the “2RS” class are freely estimated because its members use both RS as part of their response strategy. An important aspect of this transcription is that we do *not* imply that some respondents do not have ERS or MRS per se. In contrast, we imply that, even though all respondents may have any values on ERS and MRS traits, some of them might not use them on certain occasions due to the reasons mentioned in the introduction. Fixing the factor loadings of an RS to zero means that the RS does not affect the observed rating responses or, more formally, that no variance is explained by the underlying RS factor in the model.

For the further description of the MM-IRTree model, we will first introduce the response model for each class separately. The response models assume that respondents are homogeneous in their response strategy within a given class. Using the same notation as in M-IRTree (see Equation 1), Equation 2 presents the response model for the “ERS only” class, where respondents use the substantive trait and ERS as part of their response strategy. This model will be referred to as “ERS IRTree” since it only models ERS:

(2) $$\begin{array}{c} \overset{\mathrm{ERS}}{\mathrm{Pr}}\left(y_{\mathrm{pj}}|\theta_{p},\eta_{p}^{\;\mathrm{ers}},\alpha_{j}^{\;\theta },\alpha_{j}^{\;\mathrm{ers}},\omega ,\beta_{0j},\beta_{1j},\beta_{2j}\right)\\ =g^{-1}\left(y_{\;p0j}^{*}\left[-\alpha_{j}^{\;\theta }\theta_{p}-\beta_{0j}\right]\right)\\ \times g^{-1}{\left(y_{\;p1j}^{*}\left[-\alpha_{j}^{\;\theta }\theta_{p}-\beta_{1j}\right]\right)}^{I\left(y_{\mathrm{pj}}\geq 3\right)}\\ \times g^{-1}{\left(y_{\;p2j}^{*}\left[\pm \alpha_{j}^{\;\theta }\omega \theta_{p}+\alpha_{j}^{\;\mathrm{ers}}\eta_{p}^{\;\mathrm{ers}}-\beta_{2j}\right]\right)}^{I\left(y_{\mathrm{pj}}\neq 3\right)} \end{array}$$

Equation 3 presents the response model for the “MRS only” class, where respondents use the substantive trait and MRS trait as part of their response strategy. This model will be referred to as “MRS IRTree” since it only models MRS:

(3) $$\begin{array}{c} \overset{\mathrm{MRS}}{\mathrm{Pr}}\left(y_{\mathrm{pj}}|\theta_{p},\eta_{p}^{\;\mathrm{mrs}},\alpha_{j}^{\;\theta },\;\alpha_{j}^{\;\mathrm{mrs}},\omega ,\beta_{0j},\beta_{1j},\beta_{2j}\right)\\ =g^{-1}\left(y_{\;p0j}^{*}\left[-\alpha_{j}^{\;\theta }\theta_{p}-\alpha_{j}^{\;\mathrm{mrs}}\eta_{p}^{\;\mathrm{mrs}}-\beta_{0j}\right]\right)\\ \times g^{-1}{\left(y_{\;p1j}^{*}\left[-\alpha_{j}^{\;\theta }\theta_{p}+\alpha_{j}^{\;\mathrm{mrs}}\eta_{p}^{\;\mathrm{mrs}}-\beta_{1j}\right]\right)}^{I\left(y_{\mathrm{pj}}\geq 3\right)}\\ \times g^{-1}{\left(y_{\;p2j}^{*}\left[\pm \alpha_{j}^{\theta }\omega \theta_{p}-\beta_{2j}\right]\right)}^{I\left(y_{\mathrm{pj}}\neq 3\right)} \end{array}$$

Equation 4 presents the response model for the “2RS” class, where respondents use the substantive trait, ERS, and MRS as part of their response strategy. It is the same model as the one presented in the M-IRTree section, but from now on, we will refer to it as “2RS IRTree” for consistency.

(4) $$\begin{array}{c} \overset{2\mathrm{RS}}{\mathrm{Pr}}\left(y_{\mathrm{pj}}|\theta_{p},\eta_{p}^{\;\mathrm{ers}},\eta_{p}^{\;\mathrm{mrs}},\alpha_{j}^{\;\theta },\;\alpha_{j}^{\;\mathrm{mrs}},\;\alpha_{j}^{\;\mathrm{ers}},\omega ,\beta_{0j},\beta_{1j},\beta_{2j}\right)\\ =g^{-1}\left(y_{\;p0j}^{*}\left[-\alpha_{j}^{\;\theta }\theta_{p}-\alpha_{j}^{\;\mathrm{mrs}}\eta_{p}^{\;\mathrm{mrs}}-\beta_{0j}\right]\right)\\ \times g^{-1}{\left(y_{\;p1j}^{*}\left[-\alpha_{j}^{\;\theta }\theta_{p}+\alpha_{j}^{\;\mathrm{mrs}}\eta_{p}^{\;\mathrm{mrs}}-\beta_{1j}\right]\right)}^{I\left(y_{\mathrm{pj}}\geq 3\right)}\\ \times g^{-1}{\left(y_{\;p2j}^{*}\left[\pm \alpha_{j}^{\;\theta }\omega \theta_{p}+\alpha_{j}^{\;\mathrm{ers}}\eta_{p}^{\;\mathrm{ers}}-\beta_{2j}\right]\right)}^{I\left(y_{\mathrm{pj}}\neq 3\right)} \end{array}$$

Finally, Equation 5 presents the response model for the “0RS” class, where respondents use the substantive trait but neither ERS nor MRS as part of their response strategy. This model will be referred to as “0RS IRTree” since it does not model any RS.

(5) $$\begin{array}{c} \overset{0\mathrm{RS}}{\mathrm{Pr}}\left(y_{\mathrm{pj}}|\theta_{p},\alpha_{j}^{\;\theta },\omega ,\beta_{0j},\beta_{1j},\beta_{2j}\right)\\ =g^{-1}\left(y_{\;p0j}^{*}\left[-\alpha_{j}^{\;\theta }\theta_{p}-\beta_{0j}\right]\right)\\ \times g^{-1}{\left(y_{\;p1j}^{*}\left[-\alpha_{j}^{\;\theta }\theta_{p}-\beta_{1j}\right]\right)}^{I\left(y_{\mathrm{pj}}\geq 3\right)}\\ \times g^{-1}{\left(y_{\;p2j}^{*}\left[\pm \alpha_{j}^{\;\theta }\omega \theta_{p}-\beta_{2j}\right]\right)}^{I\left(y_{\mathrm{pj}}\neq 3\right)} \end{array}$$

For the MM-IRTree model, let $z_{p}\in$ {1: “ERS only,” 2: “MRS only,” 3: “2RS,” 4: “0RS”} denote the class membership of respondent *p*, and let I() be the indicator function that yields “1” if its argument is true, “0” otherwise. Using the same tree structure and pseudo-item decomposition as used previously, the response model under the MM-IRTree model is the following:

(6) $$\begin{array}{c} \overset{\mathrm{MIX}}{\mathrm{Pr}}\left(y_{\mathrm{pj}}|z_{p},\theta_{p},\eta_{p}^{\;\mathrm{ers}},\eta_{p}^{\;\mathrm{mrs}},\alpha_{j}^{\;\theta },\;\alpha_{\mathrm{jz}}^{\;\mathrm{ers}},\alpha_{\mathrm{jz}}^{\;\mathrm{mrs}},\omega ,\beta_{0\mathrm{jz}},\beta_{1\mathrm{jz}},\beta_{2\mathrm{jz}}\right)\;\\ =\left\{\overset{\mathrm{ERS}}{\mathrm{Pr}}\left(y_{\mathrm{pj}}|\theta_{p},\eta_{p}^{\;\mathrm{ers}},\alpha_{j}^{\;\theta },\;\alpha_{j1}^{\;\mathrm{ers}},\omega ,\beta_{0j1},\beta_{1j1},\beta_{2j1}\right)\times I\left(z_{p}=1\right)\right\}\\ +\left\{\overset{\mathrm{MRS}}{\mathrm{Pr}}\left(y_{\mathrm{pj}}|\theta_{p},\eta_{p}^{\;\mathrm{mrs}},\alpha_{j}^{\;\theta },\;\alpha_{j2}^{\;\mathrm{mrs}},\omega ,\beta_{0j2},\beta_{1j2},\beta_{2j2}\right)\times I\left(z_{p}=2\right)\right\}\\ +\left\{\overset{2\mathrm{RS}}{\mathrm{Pr}}\left(y_{\mathrm{pj}}|\theta_{p},\eta_{p}^{\;\mathrm{ers}},\eta_{p}^{\;\mathrm{mrs}},\alpha_{j}^{\;\theta },\;\alpha_{j3}^{\;\mathrm{mrs}},\;\alpha_{j3}^{\;\mathrm{ers}},\omega ,\beta_{0j3},\beta_{1j3},\beta_{2j3}\right)\times I\left(z_{p}=3\right)\right\}\\ +\left\{\overset{0\mathrm{RS}}{\mathrm{Pr}}\left(y_{\mathrm{pj}}|\theta_{p},\alpha_{j}^{\;\theta },\omega ,\beta_{0j4},\beta_{1j4},\beta_{2j4}\right)\times I\left(z_{p}=4\right)\right\} \end{array}$$

The MM-IRTree consists of four response models (i.e., four IRTree models) in Equations 2 to 5.^4^ Depending on the class membership of a respondent $z_{p}$ , one of these IRTree models is activated, and the rest are deactivated by the indicator function because $\sum_{c=1}^{4}I\left(z_{p}=c\right)=1$ . Therefore, depending on their class membership, the MM-IRTree allows respondents to follow a different response model.

Before going into details of the model estimation, we explain why we made some parameters class-invariant and others class-specific. First, we made factor loadings of the substantive trait ( $\alpha_{j}^{\;\theta }$ and ω ) class-invariant. Our main reason for assuming class-invariant factor loadings for the substantive trait is to ensure that we measure the same substantive trait in all classes, and classes differ from each other only due to differences in RS factor loadings. We believe that specifying class-specific $\alpha_{j}^{\theta }$ is empirically testable and can make sense when researchers do not aim to obtain trait estimates that are comparable across classes but merely aim to examine response processes. However, in this study, our aims include: (1) to find classes that differ only due to the different use of RS, (2) to have θ estimates that are comparable between latent classes and corrected for the actively used response styles in each class.

Second, we set factor loadings of RS traits $(\alpha_{\mathrm{jz}}^{\;\mathrm{ers}}$ and $\alpha_{\mathrm{jz}}^{\;\mathrm{mrs}})$ to be class-specific. That is, for instance, even though respondents in both the “ERS only” class and the “2RS” class use ERS as part of their response strategy, we allow them to use it to different extents. The reason for class-specific RS factor loadings is that the MM-IRTree is about capturing different uses of RS, which includes (1) qualitative differences regarding which RS are engaged in response strategies as implemented by fixations of RS factor loadings to zero, and (2) quantitative differences regarding how much a given RS affects the responses if it is engaged in response strategies as implemented by class-specific RS factor loadings. Nevertheless, depending on the research question, one can set the factor loadings of a RS equal across classes where this RS is used and test the feasibility of this equality constraint.

Third, we assume node difficulties to be class-specific for two reasons. The first reason is we find it unrealistic to have different numbers of traits yet the same node difficulties in different classes, and the second reason is to allow for an increase in the class separation (i.e., how easy it is to distinguish classes from each other).

Finally, we assume trait scores to be multivariate normally distributed, where their expectations are fixed at zero and variances are fixed at one for identification. However, correlations between traits are freely estimated and made class-invariant. The reason for a class-invariant correlation matrix is that we do not expect relationships between the latent variables in our model to change between classes since we ensure that the same substantive trait is measured in all classes via class-invariant $\alpha_{j}^{\theta }$ . Moreover, a common correlation matrix also keeps the model more parsimonious.

In summary, with the MM-IRTree model, we assume that all respondents have values on the substantive trait θ, the ERS trait $\eta^{\mathrm{ers}}$ , and the MRS trait $\eta^{\mathrm{mrs}}$ , but respondents differ in how they use these latent processes. That is, each of the four latent classes is associated with a different combination of RS traits to represent a distinct cognitive process, while a probabilistic IRTree model with individual person parameters and class-specific item parameters is estimated for each latent class. The latent classes thus represent theory-driven cognitive processes of response behavior that are characterized by combinations of person parameters underlying the choice of response categories, and each latent class forms a probabilistic component model in the mixture distribution framework.

In other words, the proposed MM-IRTree can be considered as a confirmatory mixture approach that combines the specification of hypothesized response processes with probabilistic component models and probabilistic assignment of respondents to latent subpopulations.

### Estimation

In the simulation study and empirical example, we also fit single-class IRTree models specified in Equations 2 to 5 in addition to the MM-IRTree as benchmarks for model selection and parameter recovery. For all models estimated in this paper, we use a Bayesian MCMC algorithm. We implemented the estimation in “Just Another Gibbs Sampler” (JAGS; Plummer, 2017). We used JAGS via the “runjags” package (Denwood, 2016) in the “R” software (R Core Team, 2021). For processing the MCMC outputs, we used the “MCMCvis” package (Youngflesh, 2018) in the R software. All of our model syntaxes, generated data sets, and the R syntax for the analysis of the empirical data set are provided on Open Science Framework and can be accessed via https://osf.io/jq36d/.

Here, we summarize our choices for prior distributions for parameter estimation. First, for the single-class 0RS IRTree model, where there is only the substantive trait as a person parameter, we specified a standard normal distribution. For the other single-class IRTree models and the MM-IRTree, we specified a multivariate normal distribution where means were fixed at zero, variances were fixed at one, and correlations between the modeled traits were freely estimated. For any correlation parameter, we specified a non-informative uniform prior *U*(−1,1). For any factor loading in all models, we specified a slightly informative normal prior truncated to be positive *N*(0, 2)*T*(0,). For any node difficulty, we specified a slightly informative normal prior *N*(0,2). For the proportionality constant ω , we specified a uniform prior *U*(0, 2). The choice for this prior distribution is based on empirical findings (Meiser et al., 2019; Merhof & Meiser, 2023). Also, different distribution types (e.g., lognormal, normal, and uniform) and ranges were tried, and there were no remarkable differences in both point estimates and posterior standard deviations (see Table S3 in Supplementary Materials). For class memberships, we specified a hierarchical prior. First, class proportions (π) are drawn from a Dirichlet distribution that is uniform in four-dimensional space. Then, the class membership of each respondent is drawn from a categorical distribution that takes the class proportions as parameters. This way, we ensure that most respondents are assigned to the classes with higher class proportions, whereas only a small number of respondents are assigned to the classes with smaller proportions. Moreover, when a class is absent in the population (i.e., $\pi_{c}=0)$ , this type of prior forces the non-existent class to stay empty in the estimation (Gelman et al., 2013; Tijmstra et al., 2018).

## Simulation Study

### Simulation Design

In this section, we describe the settings of our simulation study, which we conducted to investigate how well we can recover item and trait parameters, trait correlations, class proportions, and class memberships. In the simulation study, we manipulated the class proportions of the population model $\left\{\pi_{\mathrm{ERS}},\pi_{\mathrm{MRS}},\pi_{2\mathrm{RS}},\pi_{0\mathrm{RS}}\right\}$ . and obtained nine conditions. The population model in the first five conditions is a mixture of four latent classes with the following proportions: (1) Equal {.25, .25, .25, .25}, (2) “ERS onl dominated {.70, .10, .10, .10}, (3) “MRS only” dominated {.10, .70, .10, .10}, (4) “2RS” dominated {.10, .10, .70, .10}, (5) “0RS” dominated {.10, .10, .10, .70}. In the rest of the conditions, population models are non-mixture; in other words, only one of the classes exists in the population. We included such non-mixture conditions to examine (1) whether simpler one-class IRTree models are selected over MM-IRTree when the population is a non-mixture and (2) whether applying MM-IRTree to a non-mixture population yields satisfactory parameter estimates. The non-mixture population conditions are: (6) Single “ERS only” Class {1,0,0,0}, (7) Single “MRS only” Class {0,1,0,0}, (8) Single “2RS” Class {0,0,1,0}, (9) Single “0RS” Class {0,0,0,1} (see Table S2 in Supplementary Materials).

For each of the nine conditions, we created 10 data sets containing responses from 2,000 respondents on 20 items with a 5-point rating scale. The data sets were generated in the following steps:

1. We drew θ , *$\eta^{\mathrm{ers}}$ *, and *$\eta^{\mathrm{mrs}}$* scores from $\mathrm{MVN}\left(0,\left[\begin{array}{ccc} 1 & .20 & 0\\ \; & 1 & -.40\\ \; & \; & 1 \end{array}\right]\right)$ ^5^ for 2,000 respondents.
2. We drew the factor loadings of the substantive trait $\left(\alpha_{j}^{\;\theta }\right)$ from U(0.75,1.75) and set its proportionality constant ω to 0.50^6^ for all 20 items. The factor loadings of the ERS trait in both “ERS only” $\left(\alpha_{j1}^{\;\mathrm{ers}}\right)$ and “2RS” $\left(\alpha_{j3}^{\;\mathrm{ers}}\right)$ classes were drawn from U(0.75,1.25) , whereas the factor loadings of the MRS trait in both “MRS only” $\left(\alpha_{j2}^{\;\mathrm{mrs}}\right)$ and “2RS” $\left(\alpha_{j3}^{\;\mathrm{mrs}}\right)$ classes were drawn from U(0.50,0.75). ^7^ Regardless of the class, all item difficulties $\left(\beta_{\mathrm{jkz}}\right)$ were drawn from U(−2,2) .
3. Class memberships were generated according to the class proportion condition. The first $2,000\times \pi_{\mathrm{ERS}}$ respondents were assigned to the “ERS only” class. Then, the following $2,000\times \pi_{\mathrm{MRS}}$ respondents were assigned to the “MRS only” class. The next $2,000\times \pi_{2\mathrm{RS}}$ were assigned to the “2RS” class, while the rest were assigned to the “0RS” class.^8^
4. Depending on the class membership of a respondent, category probabilities were calculated by plugging the generated parameter values into Equations 2, 3, 4, or 5. Therefore, for each respondent, we obtained five probabilities corresponding to five response categories per item.
5. We generated multinomial responses by using the probabilities in the previous step. This step yielded a 2,000×20 matrix, where each cell takes a value between one and five.
6. The responses we generated in the previous step were decomposed into pseudo-items, as depicted in Table 1. Therefore, our final data set had 2,000 rows representing respondents and 20×3=60 columns representing pseudo-items, where each cell is zero, one, or missing, depending on the pseudo-item coding.
7. We repeated Step 5 and Step 6 to generate 10 data sets.
8. We repeated Step 4 to Step 7 for each class proportion condition.

In total, we generated 9 (conditions) ×10 (replications) =90 data sets.^9^ For each data set, we fit the MM-IRTree, ERS IRTree, MRS IRTree, 2RS IRTree, and 0RS IRTree. For the estimation of the MM-IRTree, we run ten parallel MCMC chains, each with 3,000 iterations. We discarded the first 1,000 iterations of each chain as the burn-in period, which left 20,000 iterations per data set. For the estimation of each single-class IRTree model, we run five parallel chains, each with 3,000 iterations. We discarded the first 1,000 iterations per chain as the burn-in period, which left 10,000 iterations per data set. We checked the model convergence with Rhat values for all parameters and additionally by visual inspection of the trace plots for item and class proportion parameters. The MCMC settings we used yielded Rhat values lower than 1.05 and well-mixed trace plots. We used the expectations of univariate posterior distributions as parameter estimates except for the class memberships. We explain below how we assigned respondents to classes.

### Simulation Results

#### Model Selection

It is essential to demonstrate the accuracy of model selection for the MM-IRTree model when there exists a mixture population model underlying the data. However, due to its complexity, the MM-IRTree model may overfit and incorrectly be chosen over a simpler single-class IRTree model when the data originate from a non-mixture model. Therefore, it is crucial to verify that a single-class IRTree model is accurately selected when the population model is composed of only one class. To achieve this objective, we employed the Deviance Information Criterion (DIC). The DIC is a relative fit index that assesses the model fit while also considering the model’s complexity. There are various methods of calculating the DIC, depending on the model’s characteristics (Celeux et al., 2006; Spiegelhalter et al., 2002). Our calculation of the model fit term involves the expectation of deviance across MCMC iterations $\left(\bar{D}\right)$ , while the model complexity term is calculated as half of the variance of the deviance across MCMC iterations (pV). The sum of $\bar{D}$ and pV yields the DIC. It should be noted that DIC is only meaningful when comparing multiple models, and the model with the smallest DIC is chosen as the model with the best balance of fit and parsimony.

In our simulation study, we found that across all replications of the five mixture class proportion conditions, DIC consistently selected the MM-IRTree model as the best-fitting model, and across all replications of four non-mixture conditions, DIC favored the single-class IRTree model matching with the population model (see Table 2). This highlights the utility of DIC as a reliable method for selecting the appropriate IRTree model, whether it be the MM-IRTree model for mixtures or the single-class IRTree model for non-mixture data.^10^

**Table 2.** Deviance Information Criterion and (pV) Calculated for the Last Replication of Each Condition. The Smallest Value in Each Row Is Given in Bold Face, Indicating the Chosen Model.

Class Proportions	DIC (pV)				
	MM-IRTree	ERS IRTree	MRS IRTree	2RS IRTree	0RS IRTree
**Mixtures**					
{.25, .25, .25, .25}	**94,330 (5,337)**	110,353 (4,982)	111,276 (5,098)	113,146 (9,488)	110,286 (2,290)
{.70, .10, .10, .10}	**98,195 (8,081)**	105,560 (4,515)	108,497 (5,109)	106,724 (7,269)	107,309 (2,256)
{.10, .70, .10, .10}	**96,319 (5,511)**	106,167 (4,316)	105,692 (4,376)	105,970 (6,657)	106,244 (2,292)
{.10, .10, .70, .10}	**93,282 (6,663)**	103,400 (4,710)	104,228 (4,496)	103,517 (7,220)	104,345 (2,174)
{.10, .10, .10, .70}	**94,454 (4,012)**	105,968 (4,870)	105,693 (4,592)	108,448 (8,797)	104,887 (2,188)
**Non-mixtures**					
{1, 0, 0, 0}	95,983 (5,391)	**95,226 (4,455)**	100,675 (4,664)	97,013 (6,461)	98,450 (2,266)
{0, 1, 0, 0}	97,404 (5,783)	98,492 (4,238)	**96,185 (4,368)**	98,308 (6,903)	96,924 (2,285)
{0, 0, 1, 0}	92,925 (8,156)	91,857 (4,329)	94,300 (4,368)	**91,677 (6,706)**	94,733 (2,285)
{0, 0, 0, 1}	94,930 (3,803)	96,471 (5,545)	94,944 (3,836)	97,444 (6,709)	**93,466 (2,139)**

#### Recovery of Classifications

Class proportions (π) are critical for accurate classification, as biased estimates can lead to biased class memberships. In our study, we found that the MM-IRTree successfully recovered class proportions in both mixture and non-mixture populations, with mean biases and RMSE values smaller than 0.005 (see Table S4 in Supplementary Materials for specific values).

Our approach for assigning respondents to their classes is based on using posterior class probabilities instead of the expectation of posterior distributions. This is done through the calculation of the posterior class probability for each respondent based on their response vectors, which is then used to assign the respondent to the class for which their probability is highest. This procedure is known as modal assignment (Dias & Vermunt, 2008). The posterior class probability of a respondent is calculated as the proportion of MCMC iterations in which this respondent is assigned to a given class. Then, respondents are assigned to the class for which they have the highest posterior class probability (i.e., the mode of the posterior class membership draws).

**Figure 2.**
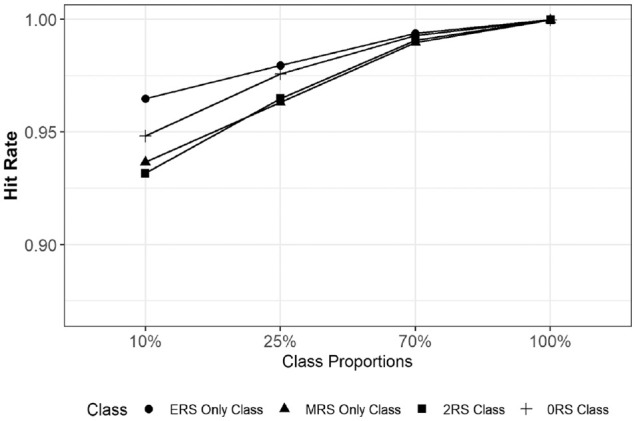
Hit Rates (Proportion of Respondents Correctly Classified Into Their True Classes) for Each Class as a Function of Class Proportions. Values Are Averaged Across Replications of a Condition.

To investigate the performance of MM-IRTree in class assignments, we calculated the proportion of respondents who are correctly assigned to their true classes (i.e., hit rate). In Figure 2, we provide the average hit rates per class averaged across the 10 replications of each condition. Overall, the MM-IRTree has very high hit rates while classifying respondents regardless of true class proportions. Hit rates were between 0.93 and 0.96 for a class proportion of 10%, 0.96 and 0.98 for 25%, and above 0.99 for 70%. Even when applied to a non-mixture population, 99.9% of respondents were assigned to their true class. Overall, MM-IRTree’s classification accuracy is very high, even for small class proportions.

#### Recovery of Item Parameters

**Figure 3.**
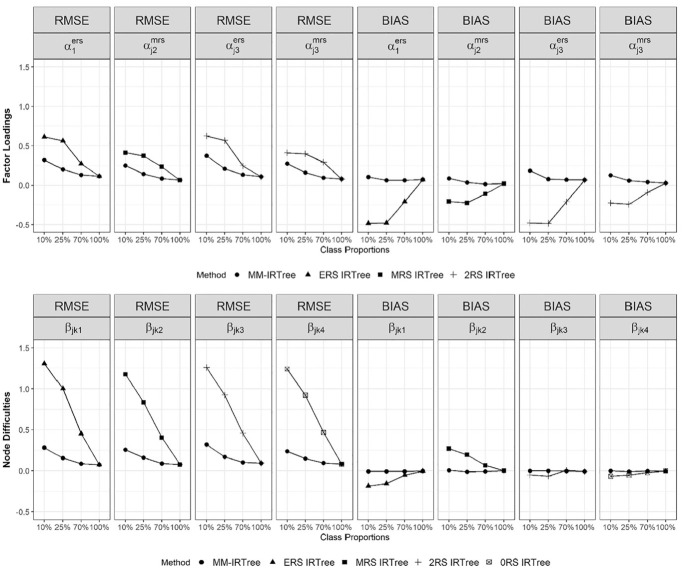
Bias and RMSE of Class-Specific Factor Loadings of Response Style Traits and Class-Specific Node Difficulties. Both Bias and RMSE are Averaged Across Replications and Items (and Nodes for Difficulties). *Note.* $\alpha_{j1}^{\mathrm{ers}}$ and $\alpha_{j3}^{\mathrm{ers}}$ are the factor loadings of ERS trait in ERS only and 2RS classes, respectively. $\alpha_{j2}^{\mathrm{mrs}}$ and $\alpha_{j3}^{\mathrm{mrs}}$ are the factor loadings of MRS trait in MRS only and 2RS classes, respectively. $\beta_{\mathrm{jk}1}$ , $\beta_{\mathrm{jk}2}$ , $\beta_{\mathrm{jk}3}$ , and $\beta_{\mathrm{jk}4}$ are the difficulty parameters (averaged across nodes and replications) in ERS only, MRS only, 2RS and 0RS classes, respectively.

For class-specific RS factor loadings and difficulty parameters, we compared the MM-IRTree model with the single-class IRTree models consisting of those specific RS factor loadings and difficulty parameters (see Figure 3). For $a_{j}^{\;\theta }$ , we discuss the findings here and in Table 3, but the findings regarding the proportionality constraint ω is discussed here only in text and the table with specific values are presented in the Supplementary Materials Table S5. We averaged the bias and RMSE of all parameters across the 20 items. In addition, we averaged the bias and RMSE of difficulty parameters across the three nodes.

**Table 3.** Bias (RMSE) of the Factor Loadings of the Substantive Trait $\left(\alpha_{j}^{\theta }\right)$ . Both Bias and RMSE Are Averaged Across Items.

Class Proportions	Estimated Model				
**Mixtures**	MM-IRTree	ERS IRTree	MRS IRTree	2RS IRTree	0RS IRTree
{.25, .25, .25, .25}	0.06 (0.09)	−0.17 (0.20)	−0.15 (0.20)	−0.15 (0.20)	−0.17 (0.20)
{.70, .10, .10, .10}	0.05 (0.08)	−0.10 (0.16)	−0.08 (0.19)	−0.07 (0.18)	−0.11 (0.17)
{.10, .70, .10, .10}	0.06 (0.09)	−0.09 (0.13)	−0.09 (0.13)	−0.08 (0.13)	−0.09 (0.13)
{.10, .10, .70, .10}	0.06 (0.09)	−0.08 (0.15)	−0.12 (0.15)	−0.11 (0.14)	−0.09 (0.15)
{.10, .10, .10, .70}	0.06 (0.09)	−0.12 (0.15)	−0.13 (0.16)	−0.14 (0.16)	−0.12 (0.15)
**Non-mixtures**					
{1, 0, 0, 0}	0.05 (0.08)	0.05 (0.08)	0.04 (0.08)	0.05 (0.08)	0.04 (0.08)
{0, 1, 0, 0}	0.06 (0.09)	0.05 (0.08)	0.05 (0.08)	0.05 (0.08)	0.05 (0.08)
{0, 0, 1, 0}	0.05 (0.09)	0.07 (0.10)	0.03 (0.08)	0.05 (0.09)	0.06 (0.11)
{0, 0, 0, 1}	0.05 (0.08)	0.04 (0.08)	0.05 (0.08)	0.05 (0.08)	0.04 (0.08)

##### Class-Specific Factor Loadings of RS

In the presence of a mixture population, the MM-IRTree slightly overestimated class-specific factor loadings of RS traits but still yielded accurate parameter estimates as evidenced by mean bias and RMSE. In contrast, single-class IRTree models led to remarkable underestimation and inaccurate parameter estimates, which were then mitigated as the proportion of classes where RS factor loadings are freely estimated increased. However, even with the largest class proportion in the mixture condition (i.e., 70%), single-class IRTree models yielded twice as large RMSE values as the MM-IRTree model, highlighting the need for using MM-IRTree when the data come from a mixture population.

When the data were not generated from a mixture population (100% condition in Figure 3), both MM-IRTree and single-class IRTrees perform comparably and yield unbiased and accurate parameter estimates.

##### Class-Specific Node Difficulties

When the data came from a mixture population model, the difficulty parameters were estimated accurately and with no systematic bias by the MM-IRTree. In contrast, single-class IRTree models yielded mixed results, tending to over- or under-estimate the difficulty parameters in addition to the inaccuracy evidenced by high RMSE values. Nevertheless, even when the largest class proportion in the mixture condition was considered (i.e., 70%), single-class IRTree models produced RMSE values that were twice as high as those obtained with the MM-IRTree model, emphasizing the need for using MM-IRTree when the data originate from a mixture population.

When the data came from a non-mixture population (100% condition in Figure 3), both the MM-IRTree and single-class IRTree models performed equivalently, providing accurate and unbiased difficulty estimates.

Results from both class-specific RS factor loadings and node difficulties show that in the presence of a mixture population, it was only the MM-IRTree that yielded accurate and unbiased parameter estimates, while single-class IRTrees provided highly unreliable estimates. In the non-mixture conditions, both MM-IRTree and single-class IRTrees performed comparably.

##### Class-Invariant Factor Loadings of the Substantive Trait

In the mixture population conditions, the MM-IRTree model tended to slightly overestimate $\alpha_{j}^{\;\theta }$ parameters in all conditions, but still with an excellent accuracy as evidenced by RMSE values (See Table 3). In contrast, fitting any of the single-class IRTree models incorrectly to the mixture data resulted in inaccurate underestimated $\alpha_{j}^{\;\theta }$ parameters. However, in the non-mixture population model conditions, both the MM-IRTree and single-class IRTree models exhibited similar bias, with a slight tendency toward overestimation.

For the proportionality constant ω , bias and RMSE results show that MM-IRTree provides accurate estimates with almost no systematic bias in both mixture and non-mixture population models. More specifically, in all conditions, mean bias and RMSE values were equal or smaller than 0.02. Similarly, single-class IRTree models provide accurate estimates despite a slight under- or overestimation in mixture population conditions. Specifically, mean biases and RMSE values were smaller than 0.10. In the non-mixture population conditions, all single-class IRTrees provide accurate estimates with very small RMSE values and almost no systematic bias (≤0.06) (see Table S5 in Supplementary Materials).

#### Recovery of Person Parameters

##### Recovery of θ

To illustrate the recovery of θ scores in the presence of a mixture population model, we used the equal class proportions condition as a representative case. For the non-mixture conditions, we compared the MM-IRTree model with the single-class IRTree model that matches with the population class. For instance, for the single “ERS only” class condition, we compared the recovery of θ scores between MM-IRTree and ERS IRTree. Wprovide the plots for only one condition because if we had included plots for nine conditions and five IRTree models, it would yield 2×9×5=90 . plots. Therefore, here, we provide results for only the equal class proportions condition and discuss the results for non-mixture conditions.

**Figure 4.**
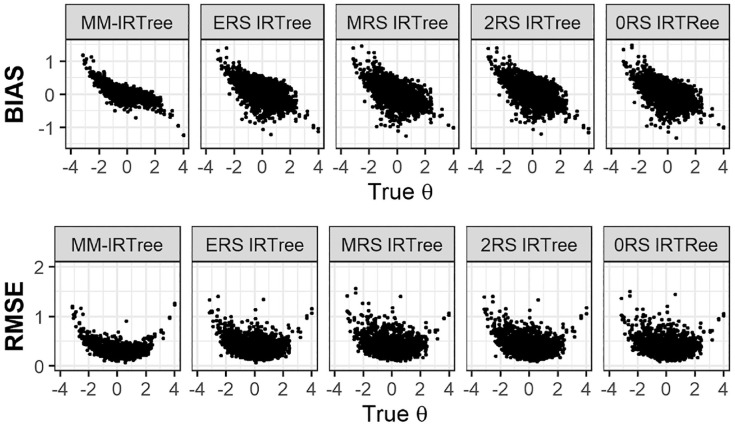
Bias and RMSE of the Substantive Trait (θ) Scores for MM-IRTree, ERS IRTree, MRS IRTree, 2RS IRTree, and 0RS IRTree in the Equal Class Proportions Condition.

Figure 4 presents the recovery statistics for the θ parameter in the mixture equal class proportions condition. The MM-IRTree model demonstrated minimal bias in estimating θ for parameter values between ±1 and shrunk the extreme values toward zero. In contrast, any of the single-class IRTree models produced substantially greater biases across the entire range of θ scores when applied incorrectly to a mixture of population data.^11^ Similar results were observed for the RMSE values. The MM-IRTree model yielded large RMSE only for extreme negative or positive parameter values, while the single-class IRTree models produced large RMSE across the entire range of parameter values. However, in non-mixture populations, the MM-IRTree performed comparably to the single-class IRTree model that matches to the existing class in the population.

To provide a summary of the recovery of θ scores in other mixture conditions, we can state that the MM-IRTree model consistently outperformed other models in terms of bias and RMSE across all mixture population conditions. More specifically, the single-class IRTree model corresponding to the dominating class in the population was the closest competitor to the MM-IRTree in most conditions, but their performance was always inferior to the performance of the MM-IRTree. Interested readers can refer to the supplementary materials for more detailed figures. (Figure S6 to S13)

##### Recovery of $\eta^{\mathrm{ers}}\mathrm{and}\;\eta^{\mathrm{mrs}}$

**Figure 5.**
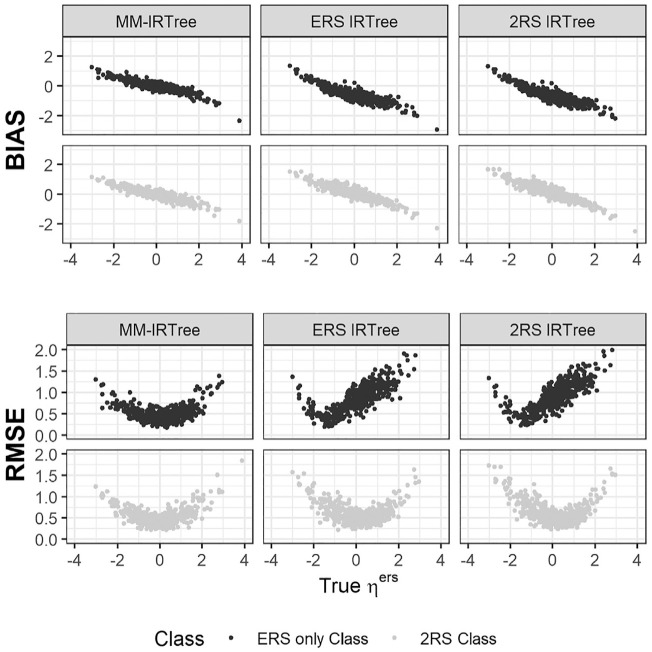
Bias and RMSE of ERS Trait Scores $\left(\eta^{\mathrm{ers}}\right)$ Obtained With MM-IRTree (Left), ERS IRTree (Middle), and 2RS IRTree (Right) Models in the Equal Class Proportions Condition.

**Figure 6.**
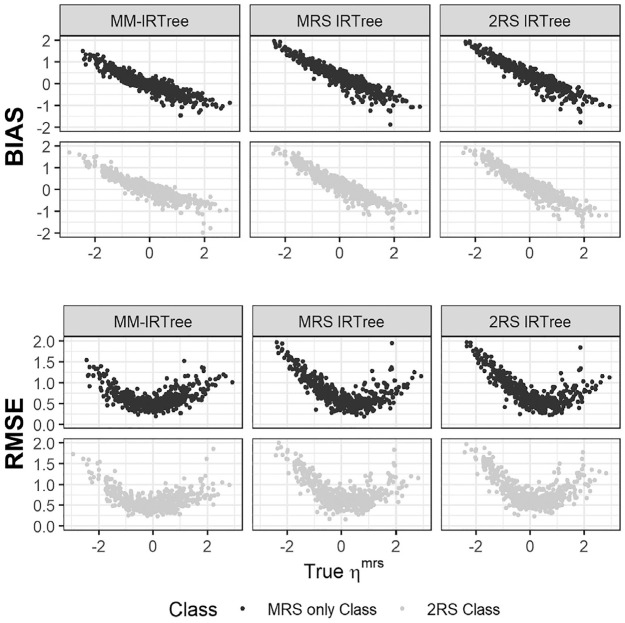
Bias and RMSE of MRS Trait Scores $\left(\eta^{\mathrm{mrs}}\right)$ Obtained With MM-IRTree (Left), MRS IRTree (Middle), and 2RS IRTree (Right) Models in the Equal Class Proportions Condition.

Regarding the recovery of RS traits, we present the results of the equal class proportions condition as a representative case of mixture population conditions. In Figure 5, we display the bias and RMSE of $\eta^{\mathrm{ers}}$ for respondents belonging to either the “ERS only” class or the “2RS” class. From the leftmost column to the rightmost column, plots show the results for the MM-IRTree, ERS IRTree, and 2RS IRTree, consecutively. In Figure 6, we present the bias and RMSE of $\eta^{\mathrm{mrs}}$ for respondents belonging to either the “MRS only” class or the “2RS” class. From the leftmost column to the rightmost column, plots show the results for the MM-IRTree, MRS IRTree, and 2RS IRTree, respectively.

An important result is that the MM-IRTree model yielded smaller bias and RMSE for the whole range of $\eta^{\mathrm{ers}}$ and $\eta^{\mathrm{mrs}}$ than single-class IRTree models in the equal class proportions condition, these findings support the reliability of the MM-IRTree in recovering RS trait scores. On the other hand, traditional single-class IRTree models performed worse than the MM-IRTree model. More specifically, they recovered RS trait scores relatively well for respondents using both ERS and MRS (Figures 5 and 6; gray dots), but they yielded biased RS trait estimates for respondents using only one type of RS (Figures 5 and 6; black dots). One might expect that the ERS scores of individuals in the “ERS only” class would be accurately recovered by the ERS IRTree model, as it is the true model for them. However, the ERS IRTree was fitted to the entire sample, which includes individuals from all four classes, rather than solely the “ERS only” subset. Therefore, the item estimates were influenced by information from individuals using MRS in the “MRS only” and “2RS” classes, resulting in more biased $\eta^{\mathrm{ers}}$ estimates for the “ERS only” class. This very same issue is also applicable to the recovery of MRS scores in the “MRS only” class using the MRS IRTree model, leading to poorer recovery due to bias introduced by the effects of the ERS users.

The poor performance of single-class IRTrees are, of course, not surprising since they were obtained from the equal class proportion condition. We expect a single-class IRTree to perform better as its matching class contains a larger proportion of respondents. Indeed, a single-class IRTree model showed better recovery when its matching class has 70% of respondents. Yet, MM-IRTree still outperformed single-class models in all mixture-class proportions conditions. In non-mixture conditions, MM-IRTree and the single-class IRTree model corresponding to the population class recovered RS traits equally well (see Supplementary Materials Figure S13 and S15).

In summary, neglecting the heterogeneity in response strategies and applying traditional single-class IRTree models to a mixture of population data resulted in biased item and trait parameters. However, the MM-IRTree model, regardless of a mixture or non-mixture population model underlying the data, provided reliable parameter estimates.

## Empirical Example

### Data Set

In this section, we illustrate the MM-IRTree model by analyzing an empirical data set from the Experiences in Close Relationships (ECR) Scale developed by Brennan and colleagues (1998). The ECR scale measures the attachment orientations of adolescents and adults. In the original study, with 36 items, authors extracted two factors named attachment anxiety (18 items, Cronbach’s alpha = 0.94) and attachment avoidance (18 items, Cronbach’s alpha = 0.91). The first factor, attachment anxiety, is characterized by the intense fear of interpersonal rejection and distress when a partner is unresponsive. A typical question measuring this factor is, “I worry a fair amount about losing my partner.” The second factor, attachment avoidance, is defined as an intense fear of interpersonal dependence and the need for self-reliance. An example item measuring this factor is, “Just when my partner starts to get close to me, I find myself pulling away.” All items in the questionnaire are rated on a 5-point scale, where categories are labeled as “Strongly disagree,”“Disagree,”“Neither agree nor disagree,”“Agree,” and “Strongly agree.”

For our empirical illustration, we use 18 items measuring attachment anxiety. The sample size of our data set is 2,000, and respondents are sampled from a larger pool with a sample size of 51,492. For choosing this subset, we first listwise excluded the respondents with at least one missing response (*N* = 4,887) and then randomly sampled 2.000 respondents. The full data set can be found online at www.openpsychometrics.org/_rawdata.

For the analysis of the data set with the MM-IRTree model, we used MCMC estimation with the Gibbs sampler implemented in JAGS. We run 20 parallel MCMC chains, each with 10,000 iterations.^12^ We discarded the first 5,000 iterations in each chain as the burn-in phase. We also estimated four single-class IRTree models for model selection purposes. For each single-class IRTree model, we run ten parallel MCMC chains, each with 10,000 iterations. The first 5,000 iterations in each chain are discarded as the burn-in phase. In total, we obtained 100,000 iterations for the estimation of MM-IRTree and 50,000 iterations per single-class IRTree model. The MCMC settings we used yielded Rhat values lower than 1.01 and well-mixed trace plots.

### Model Selection

We provide DIC values obtained for each IRTree model in Table 4.^13^ Accordingly, we chose the MM-IRTree model over single-class IRTree models as it has the smallest DIC value, in other words, the best balance of model fit $\left(\bar{D}\right)$ and complexity (pV) . Also, in the supplementary materials (Figures S4 and S5), we provide two cases of posterior predictive check indicating a good fit of MM-IRTree to the observed data.

**Table 4.** DIC (Leftmost), pV (Middle), $\bar{D}$ (Rightmost) for Single-Class IRTree Models and MM-IRTree Model in the Empirical Example. The Row With the Smallest DIC is Given in Bold Face.

Estimated Model	DIC	pV	$\bar{D}$
ERS IRTree	91,699	4,407	87,292
MRS IRTree	96,375	4,827	91,548
2RS IRTree	92,116	6,882	85,234
0RS IRTree	96,070	2,349	93,720
MM-IRTree	**87,919**	**8,250**	**79,668**

### Classifications

As the next step, we examined the estimated class proportions and classifications. As is seen in Table 5, the posterior class proportions suggest that 25% of respondents used only ERS, 15% of respondents used only MRS, 48% of respondents used both ERS and MRS, and 12% of respondents used neither ERS nor MRS as part of their response strategy in addition to the substantive trait. Regarding the classifications of respondents, 501 respondents were assigned to the “ERS only” class, 291 respondents were assigned to the “MRS only” class, 973 respondents were assigned to the “2RS” class, and 235 respondents were assigned to the “0RS” class.

**Table 5.** Estimated Class Proportions (Posterior Standard Deviations), the Number of Respondents Assigned to Each Class, and the Mean Posterior Standard Deviation of Assignments in Each Class.

Classes	π (PSD)	$N_{z}$	Certainty
ERS only	0.25 (0.01)	506	0.81
MRS only	0.17 (0.01)	333	0.74
2RS	0.48 (0.01)	986	0.86
0RS	0.10 (0.01)	175	0.75

We also calculated the classification certainty, which is the average posterior class probability across the members of a class. Overall, respondents were assigned to the larger classes (“ERS only,”“2RS”) with a higher certainty than those who belong to the smaller classes (“MRS only,”“0RS”). The observed difference in certainty between larger and smaller classes can be attributed to the availability of information for each class. Specifically, larger classes have more data points and provide greater information about the characteristics of component members, which then results in better recovery of structural parameters of a class and higher distinguishability of its members from other classes.

### Person Parameters

**Figure 7.**
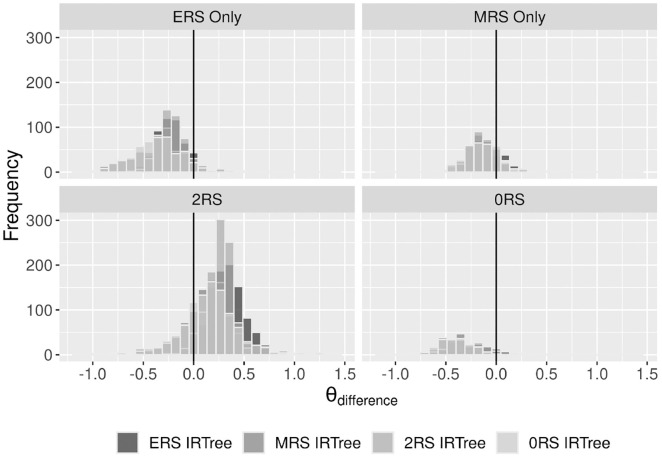
Comparison of Attachment Anxiety Scores Obtained From MM-IRTree and a Single-Class IRTree Model for Each Class. Positive $\theta_{\mathrm{difference}}$ Values Indicate MM-IRTree Yields Larger Trait Scores Than a Single-Class IRTree Model.

We were further interested in the extent to which the MM-IRTree and single-class IRTrees differ in the estimated substantive trait scores. Therefore, we calculated $\theta_{\mathrm{difference}}$ as trait scores obtained by a single-class IRTree subtracted from the ones obtained by MM-IRTree. Figure 7 shows that, compared to the MM-IRTree, all single-class IRTree models estimated attachment anxiety scores larger for respondents belonging to the ERS only, MRS only or 0RS classes and smaller for those who belong to 2RS class. A possible reason for this finding is that in single-class IRTrees, a single set of parameters was estimated with information from all classes as if they were coming from a single class. In all single-class IRTrees, the difficulties of the disagreement node for ERS only, MRS only, and 0RS classes are underestimated due to the effects of the 2RS class (2RS class has the smallest $\beta_{j0}$ , see Figure S3). This underestimation yields larger substantive trait scores estimated for these three classes, resulting in negative $\theta_{\mathrm{difference}}$ . For the 2RS class, the other three classes lead to overestimation of disagreement node’s difficulty, resulting in positive $\theta_{\mathrm{difference}}$ .

Regarding the correlations between traits, we found a positive weak correlation between attachment anxiety and ERS $\left(\sigma_{12}=0.12,\;95\%\;\mathrm{Credibility}\;\mathrm{Intervals}(\mathrm{CI})\;\left[0.04,\;0.20\right]\right)$ , a moderate positive correlation between attachment anxiety and MRS $\left(\sigma_{13}=0.28,\;95\%\;\mathrm{CI}\;\left[0.18,\;0.38\right]\right)$ , and a strong negative correlation between ERS and MRS $\left(\sigma_{23}=-0.46,\;95\%\;\mathrm{CI}\;\left[-0.55,\;-0.37\right]\right)$ . Although there is no theory or study that can explain the relationship between attachment styles and RSs, we can speculate on a broad level. First, the weak to moderate correlations between the substantive trait and RS provide suggestive evidence of discriminant validity and content irrelevancy of RS. Second, the correlation of MRS trait with attachment anxiety is stronger than of the ERS trait. This could be explained by the fact that people with a higher level of attachment anxiety tend to have lower self-esteem, and a lower self-esteem is related with having low clarity in self-concepts (Campbell, 1990). Hence, respondents with lower self-esteem might be using the middle category to provide an ambivalent response. Third, as in the earlier studies (Khorramdel et al., 2017; Nestler et al., 2021; Wetzel & Carstensen, 2017), the strong and negative correlation between ERS and MRS in our study supports that ERS and MRS are distinct but correlated dimensions rather than two ends of a single dimension.

## Conclusion and Discussion

Respondents can use RS in their response strategies as a way of heuristic responding, and thus alleviate the cost of responding (Krosnick, 1991; Krosnick et al., 1996; Podsakoff et al., 2012). Applied or methodological studies dealing with RS usually assume that respondents use all available RS (most commonly, ERS and MRS) in their response strategy. However, respondents can exhibit both ERS and MRS when there are at least five response options. Furthermore, they might choose between ERS and MRS to incorporate them into their response strategy. They can use both ERS and MRS at once, use only one of them, and use only the substantive trait to respond. Such a decision can be determined by personality attributes, cognitive skills, education level, motivation to participate, or situational factors.

Indeed, recent studies found evidence that respondents differ in what factors play a role in their response strategies. However, these studies were limited in their scope, such that the model suggested by Kim and Bolt (2021) can only be applied to 4-point rating items and neither models MRS nor the heterogeneity regarding its use, and the model suggested by Tijmstra and colleagues (2018) can be applied to only 5-point rating items and neither models ERS nor the heterogeneity in its use. The mixture IRTree model from Khorramdel and colleagues (2019) models both RS and heterogeneity in their use, but it does not allow the substantive trait to affect middle or extreme categories along with the RS. Finally, although not a mixture model, Meiser and colleagues’ (2019) model allows both the substantive trait and RS to play in response strategies (i.e., 2RS IRTree). Nevertheless, it still assumes homogeneity of response processes and may yield biased results in a population with heterogeneous response processes (e.g., poor performance of 2RS IRTree in our simulation study).

Therefore, in this study, we proposed a mixture multidimensional IRTree model that builds on and extends earlier models. The MM-IRTree approach allows us to investigate whether there are subpopulations of respondents who use (1) only ERS, (2) only MRS, (3) both ERS and MRS, and (4) no RS while the substantive trait consistently affects judgments of disagreement versus agreement, middle and extreme categories for all respondents. The benefits that the MM-IRTree framework offers are: first, it can work with 5-point items and can be extended to any number of categories. Second, it examines the simultaneous heterogeneity in the sources of middle and extreme-category responses. Third, it offers RS-corrected substantive trait scores, which incorporate individualized corrections depending on the RS set that has affected the responses. Fourth, the trait estimates can be interpreted accurately under any scale identification constraints. Fifth, the MM-IRTree provides reliable estimates of item and person parameters for researchers interested in RS research, research on the substantive trait, or scale development. Finally, the latent classes in the MM-IRTree model represent theory-guided response processes as combinations of person parameters in terms of probabilistic component models in a mixture distribution framework. Therefore, the MM-IRTree framework can also be considered as a hypothesis testing approach that tests whether all respondents indeed follow one common cognitive process involving ERS and MRS as assumed by earlier IRT models (see “Introduction” section).

Our simulation study showed that DIC can be used for model selection since it consistently favored the MM-IRTree model in the presence of a mixture population and the correct single-class IRTree in the presence of a non-mixture population. Regarding the recovery of parameters, the MM-IRTree model recovered class proportions and class memberships almost perfectly and outperformed all single-class IRTrees in the recovery of person and item parameters in the presence of a mixture population model. When the population model was a non-mixture, then MM-IRTree and single-class IRTrees performed equivalently. In summary, the MM-IRTree model was proved to be sensitive to detect true heterogeneity in response strategies and to be superior (or comparable when there is no heterogeneity) to traditional single-class IRTree approaches in estimating latent traits and item parameters.

An empirical analysis revealed that the MM-IRTree fits the best to the ECR data set, where 48% of respondents were estimated to belong to the “2RS” class, 25% to belong to “ERS only,” 15% to belong to “MRS only,” and only 12% to belong to “0RS” classes. First, these results show that the majority of respondents (88%) use at least one type of RS in their response strategy; therefore, ignoring the response style effects at all (0RS IRTree) would be the worst route to take. However, even if we were to fit a typical IRTree model containing both ERS and MRS traits (i.e., 2RS IRTree) to correct for RS effects, we would neglect 52% of respondents who follow different response strategies and, thus, obtain biased parameter estimates.

Previous studies investigating heterogeneity in RS reported that around 30% of respondents used only the substantive trait for their responses (Kim & Bolt, 2021; Tijmstra et al., 2018). A direct comparison of class proportions between different studies is not appropriate as these studies involve different populations and tests. However, we can still argue that the counterpart of “0RS” class in the earlier models may absorb respondents from the unmodelled classes and overreport the proportion who do not use any RS. Yet, a common conclusion from the current and earlier studies is that ignoring heterogeneity in response strategies has negative consequences for our inferences about the substantive trait.

We are also aware that our study has some limitations. Our simulation design is limited to one sample size (2,000) and one test length (20) condition. It would be interesting to see if the MM-IRTree performs adequately with a smaller sample size and fewer items, but given the complexity of the mixture model, we recommend using sufficiently large samples and item sets. Because the MM-IRTree performed well with 2,000 observations and 20 items, we are confident that it also works fine with larger designs.

Another limitation is that we have only nine class proportions conditions. We could have investigated even more specific cases, for example, where two out of four classes were empty and other two classes each had 50% of respondents. However, given that the MM-IRTree recovered class proportions with almost no bias in all present conditions, we are confident that it performs well with different class proportions that were not included in our study.

The last limitation to mention is that we have only ten replications per condition. The reason for keeping the number of replications small is the duration of estimation of our models. That is, even with a powerful computer, one replication that includes the estimation of the MM-IRTree model and four single-class IRTrees plus post-processing of results took around 10 hours. The computation time itself may be a limiting factor for applied fields at present, but with the increasing accessibility of high-speed computers, this limitation will diminish in the future.

In addition to accounting for our limitations, future research can further investigate the heterogeneity in response strategies in several ways. First, it would be interesting to examine predictors of class memberships. If researchers can show that class memberships are predicted by some personality attributes or cognitive skills, we can better understand the grounds of individual differences in RS compared to some existing correlational studies.

Second, future research can investigate the consistency of response strategies over time and item content. Earlier studies showed that the response strategies that people use dynamically change throughout the questionnaire (Merhof & Meiser, 2023) and across different psychological constructs (Wetzel et al., 2013). It would be interesting to investigate with the MM-IRTree whether respondents differ in their response strategies across constructs (with a second-level latent class analysis as in Wetzel et al., 2013) or over time (with an item-level mixture model as in Plieninger & Heck, 2018; Tijmstra et al., in press).

Finally, we used respondents who have a complete response vector. However, it is possible that some classes have different tendencies to skip items. For instance, the “2RS” class might be more inclined to skip items and alleviate the cognitive burden to the extent that they have stronger heuristic tendencies (i.e., satisficing, Krosnick, 1991). This would create a missing not-at-random mechanism in full-information maximum likelihood estimation (i.e., one-step estimation) or its Bayesian equivalents, resulting in biased parameter estimates (Alagöz & Vermunt, 2022). Therefore, further research can investigate whether the tendency to provide missing responses can be explained by class memberships.

In conclusion, our simulation study and empirical analysis showed that the MM-IRTree model is a valuable extension of psychometric toolbox to analyze sources of heterogeneity in response processes and to adjust trait measurements for biasing RS effects after taking the heterogeneity into account. The MM-IRTree model’s capabilities and benefits open new avenues for research and have the potential to contribute to a wide range of disciplines and applications in the social sciences.

## Supplemental Material

sj-docx-1-epm-10.1177_00131644231206765 – Supplemental material for Investigating Heterogeneity in Response Strategies: A Mixture Multidimensional IRTree Approach Supplemental material, sj-docx-1-epm-10.1177_00131644231206765 for Investigating Heterogeneity in Response Strategies: A Mixture Multidimensional IRTree Approach by Ö. Emre C. Alagöz and Thorsten Meiser in Educational and Psychological Measurement

## Notes

1. The M-IRTree model can be set up in different ways. For instance, decisions can be specified as: “Agreement,”“Neutrality,” and “Extremity.” Then, the “Neutrality” node would be nested in the “Agreement” node, and the “Extremity” node would remain distinct at the end. Such modifications would alter the node specifications. Therefore, compared to the current tree structure, some parameters may have different estimates (e.g., inverted signs of the node difficulties). However, the meaning of the model as a whole would remain identical.

2.
ω serves as a means of achieving parsimony and facilitating the interpretation of results. This concept was first introduced by Meiser et al. (2019). They found that the impact of θ on the decision between relative (dis)agreement categories was weaker (around one-third) than that on the disagreement vs. agreement decision.

3. In multidimensional 2PL models, having multiple discrimination parameters prevents us from interpreting β parameters as difficulty parameters as in unidimensional 2PL IRT models. In our model, these terms represent intercepts as in factor analysis models but with a negative sign.

4. One can think that the 2RS IRTree model would adequately account for this kind of heterogeneity in response strategies by estimating RS trait scores as zero for those who do not use it. However, such approximation to zero is only possible when a score of zero is included in the scale identification constraints such as *N*(0,1). But then, a score of zero would imply an average RS behavior, not a non-use of it. In fact, simulation results further showed that 2RS IRTree did not estimate RS trait scores close to zero for those who do not use them but rather systematically under- or overestimated them. Readers are referred to supplementary materials for a thorough discussion.

5. Our choices of correlations between traits are based on previous empirical findings. Namely, ERS and MRS are negatively correlated with each other, whereas they can have weak correlations with substantive traits (Khorramdel et al., 2017; Nestler et al., 2021; Wetzel & Carstensen, 2017).

6. This choice is based on an empirical finding (Meiser et al., 2019).

7. There is a consistent finding across several studies that ERS has a large variance compared to the MRS trait (Ames & Myers, 2021; Böckenholt & Meiser, 2017; Henninger & Meiser, 2020b; Meiser et al., 2019). We aimed to imitate those findings, but since we fixed the trait variances at one, we generated ERS factor loadings to be larger than MRS factor loadings.

8. By generating class memberships in a deterministic way, we did not allow sampling variation for them since there is only a small number of replications per condition.

9. The reason for limiting our simulation study to a fixed sample size and number of items as well as generating 10 data sets per condition, is the estimation time of one condition. The MM-IRTree model (~4 hours) and four single-class IRTree models (~6 hours), including post-processing, took around 10 hours with a computer with the following specifications: AMD Ryzen Threadripper 3970X 32-Core 64-threads Processor, 3.7 GHz base clock, and 66 GiB System memory.

10. The fact that 2RS was never chosen over MM-IRTree even in the condition where the “2RS” was dominating yields another evidence that they are not equivalent models, and that 2RS IRTree cannot account for this type of heterogeneity in response strategies.

11. Referring to footnote 5, this was another evidence proving that 2RS IRTree cannot adequately account for heterogeneity in response strategies, as it fails to recover θ scores reliably.

12. The reason for using such a high number of MCMC chains is to deal with the between-chains label-switching problem. The procedure we follow to deal with this problem is explained in the Supplementary Materials.

13. In response to an anonymous reviewer, we also estimated a modified version of the MM-IRTree, where none of the RS factor loadings were fixed at zero. Rather, we freely estimated RS factor loadings in all classes and decided on the number of classes via model selection. Results showed that, up to three classes, this modified MM-IRTree showed a worse fit than confirmatory MM-IRTree, and with four and five classes, it had a comparable fit with confirmatory MM-IRTree. Given the difficulties in interpretations, the confirmatory MM-IRTree was much more favorable. We do not discuss this modified model further as it is not in the scope of this study.
