# Supplementary material for: Investigating Heterogeneity in Response Strategies: A Mixture Multidimensional IRTree Approach
Source: Educ Psychol Meas. 2023 Nov 9;84(5):957–93. doi: 10.1177/00131644231206765 (PMC11418595; doi:10.1177/00131644231206765)

# **Supplementary Material**

## 1. Comparison Between MM-IRTree and 2RS IRTree for the Recovery of Unused RS Estimates

As mentioned briefly in Kim and Bolt (2021) that 2RS IRTree, originally proposed by Meiser et al., (2019), can adequately account for differential use of RS. That is, 2RS IRTree will approximate a RS trait estimate to zero when the respondent does not use that RS in their response strategy. Then a RS trait estimate that is equal or close to zero would imply no or negligible effect of RS in the response behaviour. Although it is theoretically reasonable, we believe that there are at least three limitations to use 2RS IRTree for this purpose.

First, when we use the traditional zero and one constraints on expectations and variances, we imply that trait scores follow a normal distribution where zero represents the average trait level. Then a RS trait estimate of zero would practically has no effect on response but interpreted as average level of RS behaviour. For instance, an ERS score of “0” still implies a higher tendency to choose extreme categories compared to an ERS score of “-1”. In this case, we can no longer distinguish a respondent with average RS level from a respondent who do not use that RS. Therefore, 2RS IRTree cannot account for non-use of a RS as it contradicts with the elementary interpretation of trait distributions and identification constraints.

Second, one can offer using a different identification constraint to solve the first problem, which is problematic in another way. That is, if researchers opt for scale identification constraint other than latent expectations of zero and variances of one (e.g., expectation of 100 and variance of 15), zero can be practically out of range or on the extreme tails of the distribution. This would prevent 2RS IRTree from approximating RS estimates to zero and cause 2RS IRTree to yield biased parameter estimates.

Third, 2RS IRTree did not approximate RS trait scores to zero for respondents who do not use them. We present a comparison between MM-IRTree and 2RS IRTree regarding the ERS trait estimates for respondents who do not use ERS (MRS only and 0RS classes), and MRS trait estimates for respondents who do not use MRS (ERS only and 0RS classes) in Figure S1. Presented results are aggregated over five mixture class proportion conditions. In Figure S1, it is clear that 2RS IRTree fails to approximate non-used RS trait scores to zero. Instead, 2RS IRTree tends to yield negative ERS scores for those who do not use ERS and positive MRS scores for those who do not use MRS. Additionally, RS trait estimates also show dispersion. A RS trait score for a person who does not use that RS, 2RS IRTree yield a score close to “-1” or “1”, which is remarkably further away from zero. On the other hand, MM-IRTree tends to yield a RS score of zero for those who do not use that RS with some dispersion. Since MM-IRTree also fixes unused RS factor loadings at zero, this dispersion does not have any implication as these RS scores do not affect response probabilities.

Combining the abovementioned drawbacks with the findings in the main text (see “Recovery of $\theta$” and Figure 5) proving that 2RS IRTree yield quite biased $\theta$ estimates when applied to a mixture population, we are confident in the added value of MM-IRTree in accounting for heterogeneity in response strategies.

[Insert Figure S1 here]

## 2. Results for the Item Parameters from Empirical Data Analysis

We were also interested in how item parameters differ between classes. In Figure S2, we present the estimated class-specific RS factor loadings and the factor loadings of the substantive trait.

First, we found that ERS factor loadings are much greater than MRS factor loadings, which implies that the ERS trait has a stronger impact on observed responses than the MRS trait. This finding is in line with previous studies and our choice of data-generating parameters in the simulation study.

Second, we found that ERS and MRS factor loadings are slightly larger in the ”2RS” class than in the “ERS only” and “MRS only” classes. An intuitive explanation could be that respondents in “2RS” class use both RS because they have a stronger tendency to respond heuristically. Moreover, this stronger tendency might further reflect on the extent they make use of a RS compared to the classes with lower heuristic tendencies who use only one RS. However, these findings and our interpretation should be taken cautiously as we did not hypothesize any difference between classes a priori, but rather made an exploratory comparison afterwards.

Third, factor loadings of the substantive trait are between 0.70 and 2.26, with a mean of 1.34. Therefore, most items seem to well discriminate between respondents with high and low levels of attachment anxiety. Finally, the proportionality constant $\omega$ is 0.70, which means that the substantive trait also influences the decision at the extremity nodes. However, since $\omega$ is smaller than one, the effect of the substantive trait at the extremity node is smaller than its effect at the disagreement and neutrality nodes. This finding means that the substantive trait affects fine-grained choices within the subset of agreement and disagreement categories, though with lower strength than the general decision between agreement versus disagreement. A weaker effect of the substantive trait at later nodes is quite reasonable as deciding between disagreement, neutrality and agreement involves a bigger contrast (different response directions) than deciding on to what extent one (dis-)agrees (same response direction). Considering other studies that are in line with our findings, there is accumulating evidence in favour of substantive trait’s effects above and beyond the response direction (Meiser et al., 2019).

[Insert Figure S2 here]

In Figure S3, we present the class-specific item difficulties for the disagreement, neutrality, and extremity nodes. At the disagreement node, most items have positive difficulty parameters in all classes. This means that respondents with average $\theta$ scores are less likely to disagree with the item content (for respondents with average $\eta^{mrs}$ in “2RS” and “MRS only” classes). Moreover, items show relatively smaller difficulty in the “2RS” class, implying that these respondents are relatively more likely to disagree with the item content.

At the neutrality node, almost all items have positive difficulty parameters in all classes, indicating that respondents with average $\theta$ scores are more likely to agree with the item content instead of staying neutral (for respondents with an average $\eta^{mrs}$ score in “2RS” and “MRS only” classes). Relatively smaller difficulties in the “2RS” class imply that respondents in this class are slightly more inclined to choose the middle category.

At the extremity node, items have positive difficulty parameters in all classes, indicating that respondents with average $\theta$ scores tend to choose an intermediate category (“2” or “4”) rather than an extreme category (for respondents with an average $\eta^{ers}$ score in “2RS” and “ERS only” classes).

[Insert Figure S3 here]

In conclusion, the MM-IRTree model does not only capture qualitative differences in response strategies (i.e., fixations of RS factor loadings to zero) but also detects quantitative differences in how RS affect responses (i.e., class-specific factor loadings of a RS given that it is engaged). Accordingly, we found in the empirical data analysis that the impacts of ERS and MRS traits on responses were stronger in the “2RS” class compared to the “ERS only” or “MRS only” classes. The reason for these findings could be that respondents using both RS have a stronger heuristic tendency than those who use only one RS; therefore, they also use both ERS and MRS to a larger extent.

## 3. Posterior Predictive Checks

For assessing the global fit of our model, we used posterior predictive check (PPC), which is a Bayesian method to assess fit of the model to the data. We briefly describe our procedure:

1. In ith iteration of MCMC, draw parameter estimates $\lambda_{(i)}$ from the joint posterior $P\left( \lambda| Y^{obs} \right)$.
2. With $\lambda_{(i)}$, generate a hypothetical data set from the sampling distribution of the model $P\left( Y_{\left( i \right)}^{rep} | \lambda_{\left( i \right)},M \right)$ where M stands for the model of interest.
3. We investigate two aspects of these hypothetical data sets; person sum scores and item sum scores. Therefore, in each $Y_{\left( i \right)}^{rep}$, we calculate person sum scores denoted by $PS_{\left( i \right)}^{rep}$and item sum scores denoted by $IS_{\left( i \right)}^{rep}$. The same are calculated also for the observed data and denoted by $PS^{obs}$ and $IS^{obs}$.
4. Then, we compare $PS^{obs}$ and $IS^{obs}$ with the average $PS^{rep}$ and $IS^{rep}$ across MCMC iterations.

In Figure S4 and S5, we provide the comparison between replicated and observed person sum scores (for each class separately) and item sum scores. Figure S4 shows that average person sum scores obtained across replicated data sets indeed are very close to observed data set, as indicated by their proximity to the diagonal line. Replicated data sets resemble the original data set better in “ERS only” and “2RS” classes, but this is an expected result as these classes contain more respondents (therefore, more information). Figure S5 shows that replicated item sum scores also are very close to the observed item sum scores. Combined, these figures support that the model fit is satisfactory.

## 4. Recovery of Person Parameters

### 4.1 $\boldsymbol{\theta}$ scores

Here, we provide additional details on the recovery of $\theta$ parameters. In the main text, we only provided results for equal class proportions conditions, where MM-IRTree outperformed the single class IRTree models. Whenever a class consists of 70% of the observations, we expect that the single class IRTree model corresponding to the dominating class will perform better regarding the recovery of $\theta$ compared to when it consists of 25% or 10% of the sample. However, when the population model underlying the data is a non-mixture, we expect MM-IRTree and the single-class IRTree corresponding to the class in the population to perform equivalently. To illustrate this, we provide the results for each mixture condition from Figure S6 to Figure S9, and each non-mixture condition from Figure S10 to Figure S13. In the text, we limit ourselves for discussing only the condition where 2RS only class has 70% of the respondents, whereas each of the rest has only 10% (Figure S8).

According to both bias and RMSE, MM-IRTree still shows the best recovery of $\theta$. As we expected there is a remarkable improvement for the results obtained with 2RS IRTree compared to the conditions where “2RS” class had smaller class proportions. However, $\theta$ estimates are still biased to some extent when 2RS IRTree is used because of the remaining 30% of respondents belonging to other classes.

In summary, in the presence of a mixture population, MM-IRTree outperforms traditional single-class IRTrees in estimating the substantive trait. The single-class IRTrees show better performance as the proportion of the corresponding class increases. The performance of MM-IRTree and single-class IRTrees become comparable in the presence of a non-mixture population. Although we provided example for 2RS IRTree and 70% “2RS” class condition, results are generalizable to other single-class IRTree approaches and to other classes.

### 4.2 $\boldsymbol{\eta}^{\boldsymbol{ers}}$ and $\boldsymbol{\eta}^{\boldsymbol{mrs}}$ scores

Here, we provide the results for the recovery of RS traits in the 2RS dominated class condition. For the recovery of $\eta^{ers}$ trait with single-class IRTrees, we see a great improvement from equal class proportions condition to 2RS dominated class condition (Figure S14). That is, both ERS IRTree and 2RS IRTree models perform very close to the MM-IRTree model. We see a similar trend for the recovery of $\eta^{mrs}$ with single-class IRTrees (Figure S15). Both the bias and RMSE of MRS IRTree and 2RS IRTree show improvement from equal classes condition to 2RS dominated class condition, but the MM-IRTree model still visibly outperforms single-class IRTree models.

## 5. Recovery of Trait Correlations

In Table S1, we present the bias and RMSE of trait correlations. When the population model is a mixture, MM-IRTree estimated correlations between all traits almost perfectly. When the population model was non-mixture, MM-IRTree always overestimated the correlation for trait pairs in which one of the traits is not used by respondents (e.g., the correlation between $\eta^{mrs}$ and $\theta$ in the single ERS only class condition).

2RS IRTree shows the worst performance for all correlation pairs except in the condition where the “2RS” class has the most respondents. In non-mixture conditions, 2RS IRTree performed well only in the single 2RS class condition. We do not discuss the results for other single-class IRTree models in mixture conditions because they can only estimate the correlation between $\theta$ and one RS. For non-mixture conditions, when the population class matches with the single class IRTree model, their performance in the recovery of a correlation is equal or only slightly worse than the MM-IRTree model.

## 6. Procedure Followed for Correcting Label Switching

One might wonder why we did not follow some well-known remedies for the between-chain label-switching issue, namely 1) running a single chain, 2) relabelling after estimation, and 3) setting initial parameter values close to their true values.

The solutions 1 and 2 do not work in confirmatory mixture models where a parameter is freely estimated in one class and fixed to zero in another class. Because, when a class with freely estimated loadings is labelled as the class for which loadings are fixed at zero, MCMC does not draw values for that parameter; rather, it assigns zero. Therefore, if we run a single chain, we cannot ensure that labels of classes with constraints and with freely estimated parameters are not switched. Relabelling after estimation is also not possible as the information for the class where RS is used but fixed at zero due to label switch is lost as MCMC assigned zero to it. The last solution is also infeasible for empirical data analysis as we do not know the true parameter values.

Therefore, in our case, the only doable solution was estimating single-class IRTrees in each class within each MCMC chain and subsequently identifying the chain where single-class IRTrees fit the best for their matching class (see the procedure below).

1. Start by running MCMC estimation with 20 chains.
2. For the first chain, follow steps 3 to 6.
3. Assign respondents into classes using the modal assignment rule.
4. Estimate all four single-class IRTree models in each class.
5. For each class, use DIC to determine which single-class IRTree model fits best.
6. If a single-class IRTree model is the best fitting model in its corresponding class, label switching did not occur. However, if a single-class IRTree fits best in another class, say, ERS IRTree in the MRS-only class, it is flagged as a potential label-switch.
7. Repeat steps 3 to 6 for the remaining MCMC chains.
8. The chain in which each single-class IRTree model is the best fitting model in their corresponding class is considered free of label switching.
9. Use the posterior estimates of the chosen chain as initial values for a new MCMC estimation, which is required to investigate convergence of MCMC and make statistical inferences from a larger pool of posterior draws.

With simulated data sets, we first tested the effectiveness of this approach. Note that a large sample size is required for this approach to work well and it might not be feasible for smaller sample sizes and different modelling approaches. Also note that, given the model selection with DIC and posterior predictive checks are made prior to this step and these statistics are not affected by label switching (i.e., invariant across chains), this approach does not pose a threat of overfitting.

# **References**

Kim, N., & Bolt, D. M. (2021). A mixture IRTree model for extreme response style: Accounting for response process uncertainty. *Educational and Psychological Measurement, 81(1),* 131-154.

Meiser, T., Plieninger, H., & Henninger, M. (2019). IRTree models with ordinal and multidimensional decision nodes for response styles and trait‐based rating responses*. British Journal of Mathematical and Statistical Psychology, 72(3)*, 501–516. <https://doi.org/10.1111/bmsp.12158>

Merhof, V., & Meiser, T. (2023). Dynamic response strategies: Accounting for response process heterogeneity in IRTree decision nodes. *Psychometrika*, 1-27.

## Tables

**Table S1**

*Bias (RMSE) of the trait correlations.*

| Conditions | MM-IRTree | | | ERS IRTree | MRS IRTree | 2RS IRTree | | |
| --- | --- | --- | --- | --- | --- | --- | --- | --- |
| **Mixtures** | $\theta,\eta^{ers}$ | $\theta,\eta^{mrs}$ | $\eta^{ers},\eta^{mrs}$ | $\theta,\eta^{ers}$ | $\theta,\eta^{mrs}$ | $\theta,\eta^{ers}$ | $\theta,\eta^{mrs}$ | $\eta^{ers},\eta^{mrs}$ |
| {.25, .25, .25, .25} | -0.02 (0.03) | 0.02 (0.04) | 0.05 (0.06) | -0.27 (0.27) | 0.01 (0.04) | -0.32 (0.32) | 0.01 (0.05) | 0.68 (0.69) |
| {.70, .10, .10, .10} | -0.01 (0.01) | 0.12 (0.13) | -0.05 (0.08) | -0.11 (0.11) | -0.05 (0.07) | -0.17 (0.17) | 0.12 (0.07) | 0.69 (0.70) |
| {.10, .70, .10, .10} | -0.02 (0.05) | 0.04 (0.05) | 0.01 (0.06) | -0.18 (0.18) | 0.05 (0.06) | -0.26 (0.26) | 0.07 (0.07) | 0.89 (0.89) |
| {.10, .10, .70, .10} | 0.01 (0.02) | 0.03 (0.04) | 0.01 (0.03) | -0.07 (0.07) | 0.07 (0.07) | -0.03 (0.04) | 0.03 (0.05) | 0.13 (0.14) |
| {.10, .10, .10, .70} | -0.02 (0.06) | 0.04 (0.07) | -0.02 (0.08) | -0.16 (0.20) | 0.12 (0.13) | -0.18 (0.18) | 0.12 (0.13) | 0.15 (0.45) |
| **Non-mixtures** |  |  |  |  |  |  |  |  |
| {1, 0, 0, 0} | 0.01 (0.02) | 0.38 (0.51) | 0.72 (0.76) | 0.01 (0.02) | 0.13 (0.20) | 0.01 (0.01) | 0.02 (0.17) | 0.43 (0.44) |
| {0, 1, 0, 0} | -0.41 (0.49) | 0.02 (0.05) | 0.35 (0.45) | -0.18 (0.19) | 0.02 (0.04) | -0.23 (0.24) | 0.02 (0.05) | 0.36 (0.37) |
| {0, 0, 1, 0} | -0.01 (0.02) | 0.03 (0.04) | -0.01 (0.02) | -0.07 (0.07) | 0.08 (0.09) | -0.01 (0.02) | 0.03 (0.04) | 0.01 (0.01) |
| {0, 0, 0, 1} | 0.03 (0.25) | 0.19 (0.38) | 0.35 (0.51) | -0.16 (0.17) | -0.03 (0.12) | -0.15 (0.16) | -0.03 (0.12) | 0.32 (0.39) |

**Table S2**

*Conditions in the simulation study.*

| Conditions | Labels | Class Proportions | | | |
| --- | --- | --- | --- | --- | --- |
| ${\{\pi}_{ERS,} \pi_{MRS}, \pi_{2RS}, \pi_{0RS}\}$ |  | ERS only | MRS only | 2RS | 0RS |
| {.25, .25, .25, .25} | Equal Classes | 25% | 25% | 25% | 25% |
| {.70, .10, .10, .10} | ERS dominated | 70% | **10%** | **10%** | **10%** |
| {.10, .70, .10, .10} | MRS dominated | **10%** | 70% | **10%** | **10%** |
| {.10, .10, .70, .10} | 2RS dominated | **10%** | **10%** | 70% | **10%** |
| {.10, .10, .10, .70} | 0RS dominated | **10%** | **10%** | **10%** | 70% |
| {1, 0, 0, 0} | Single ERS only | 100% | - | - | - |
| {0, 1, 0, 0} | Single MRS only | - | 100% | - | - |
| {0, 0, 1, 0} | Single 2RS only | - | - | 100% | - |
| {0, 0, 0, 1} | Single 0RS only | - | - | - | 100% |

*Note.* Bold faced class proportions in each column are merged for some of the results.

**Table S3**

*Prior distributions of parameters for a given IRTree model.*

| Parameters | Models | | | | |
| --- | --- | --- | --- | --- | --- |
|  | MM-IRTree | ERS IRTree | MRS IRTree | 2RS IRTree | 0RS IRTree |
| $\theta$ | - | - | - | - | $N(0,1)$ |
| $\theta,\eta^{ers}$ | - | $\mathrm{MVN}\left( \mathbf{0},\left[ \begin{matrix} 1 & \sigma_{12} \\ & 1 \end{matrix} \right] \right)$ | - | - | - |
| $\theta,\eta^{mrs}$ | - | - | $\mathrm{MVN}\left( \mathbf{0},\left[ \begin{matrix} 1 & \sigma_{13} \\ & 1 \end{matrix} \right] \right)$ | - | - |
| $\theta,\eta^{ers},\eta^{mrs}$ | $\mathrm{MVN}\left( \mathbf{0},\left[ \begin{matrix} 1 & \sigma_{12} & \sigma_{13} \\ & 1 & \sigma_{23} \\ & & 1 \end{matrix} \right] \right)$ | - | - | $\mathrm{MVN}\left( \mathbf{0},\left[ \begin{matrix} 1 & \sigma_{12} & \sigma_{13} \\ & 1 & \sigma_{23} \\ & & 1 \end{matrix} \right] \right)$ | - |
| $\alpha_{j}^{\theta},\alpha_{jz}^{ers}, \alpha_{jz}^{mrs}$ | $N\left( 0,2 \right)T\left( 0, \right)$ | $N\left( 0,2 \right)T\left( 0, \right)$ | $N\left( 0,2 \right)T\left( 0, \right)$ | $N\left( 0,2 \right)T\left( 0, \right)$ | $N\left( 0,2 \right)T\left( 0, \right)$ |
| $\beta_{jkz}$ | $N\left( 0,2 \right)$ | $N\left( 0,2 \right)$ | $N\left( 0,2 \right)$ | $N\left( 0,2 \right)$ | $N\left( 0,2 \right)$ |
| $\omega$*^1^* | $U(0,2)$ | $U(0,2)$ | $U\left( 0,2 \right)$ | $U\left( 0,2 \right)$ | $U\left( 0,2 \right)$ |
| $\sigma_{12},\sigma_{13},\sigma_{23}$ | $U\left( -1,1 \right)$ | $U\left( -1,1 \right)$ | $U\left( -1,1 \right)$ | $U\left( -1,1 \right)$ | - |
| $z$ | $\mathrm{Categorical}\left( \pi\right)$ | - | - | - | - |
| $\pi$ | $\mathrm{Dirichlet}\left( 1,1,1,1 \right)$ | - | - | - | - |

*Note.* A cell with ‘-‘ indicates that the parameter is not estimated with the IRTree model in that column.

^1^ The choice for this prior distribution is based on empirical findings (Meiser et al., 2019; Merhof & Meiser, 2023). Also, different distribution types (e.g., lognormal, normal, uniform) and ranges were tried, and there were no remarkable differences in both point estimates and posterior standard deviations

**Table S4**

*Bias (RMSE) of the class proportion* $\left( \pi\right)$ *estimates obtained with MM-IRTree.*

| Class Proportions | Classes | | | |
| --- | --- | --- | --- | --- |
| **Mixtures** | ERS only Class | MRS only Class | 2RS Class | 0RS Class |
| {.25, .25, .25, .25} | 0.001 (0.002) | 0.001 (0.002) | -0.001 (0.004) | 0.001 (0.002) |
| {.70, .10, .10, .10} | 0.001 (0.002) | 0.001 (0.002) | -0.001 (0.002) | -0.001 (0.002) |
| {.10, .70, .10, .10} | 0.001 (0.001) | 0.003 (0.004) | -0.001 (0.002) | -0.002 (0.003) |
| {.10, .10, .70, .10} | -0.003 (0.003) | 0.001 (0.002) | -0.001 (0.002) | 0.003 (0.003) |
| {.10, .10, .10, .70} | 0.001 (0.002) | 0.001 (0.002) | -0.001 (0.001) | -0.001 (0.002) |
| **Non-mixtures** |  |  |  |  |
| {1, 0, 0, 0} | -0.001 (0.002) | 0.001 (0.001) | -0.001 (0.001) | 0.001 (0.001) |
| {0, 1, 0, 0} | 0.001 (0.001) | -0.002 (0.002) | 0.001 (0.001) | 0.001 (0.001) |
| {0, 0, 1, 0} | 0.001 (0.001) | 0.001 (0.001) | -0.001 (0.001) | -0.001 (0.001) |
| {0, 0, 0, 1} | 0.001 (0.001) | 0.001 (0.001) | 0.001 (0.001) | -0.001 (0.001) |

**Table S5**

*Bias (RMSE) of the proportionality constraint* $\left( \omega\right)$*.*

| Class Proportions | Estimated Model | | | | |
| --- | --- | --- | --- | --- | --- |
| **Mixtures** | MM-IRTree | ERS IRTree | MRS IRTree | 2RS IRTree | 0RS IRTree |
| {.25, .25, .25, .25} | 0.01 (0.01) | 0.02 (0.01) | -0.05 (0.01) | -0.06 (0.01) | -0.05 (0.01) |
| {.70, .10, .10, .10} | -0.01 (0.01) | 0.02 (0.01) | -0.06 (0.01) | -0.09 (0.01) | -0.05 (0.01) |
| {.10, .70, .10, .10} | -0.01 (0.01) | 0.02 (0.01) | -0.03 (0.01) | -0.03 (0.01) | -0.05 (0.01) |
| {.10, .10, .70, .10} | -0.01 (0.01) | 0.02 (0.01) | -0.05 (0.01) | -0.04 (0.01) | -0.08 (0.01) |
| {.10, .10, .10, .70} | -0.01 (0.01) | 0.02 (0.01) | -0.01 (0.01) | 0.01 (0.01) | -0.02 (0.01) |
| **Non-mixtures** |  |  |  |  |  |
| {1, 0, 0, 0} | -0.01 (0.01) | 0.02 (0.01) | -0.03 (0.01) | -0.01 (0.01) | -0.04 (0.01) |
| {0, 1, 0, 0} | 0.01 (0.01) | 0.02 (0.01) | 0.01 (0.01) | 0.001 (0.01) | -0.01 (0.01) |
| {0, 0, 1, 0} | -0.02 (0.01) | 0.02 (0.01) | -0.04 (0.01) | -0.01 (0.01) | -0.06 (0.01) |
| {0, 0, 0, 1} | -0.01 (0.01) | 0.02 (0.01) | -0.01 (0.01) | -0.01 (0.01) | -0.01 (0.01) |

## Figures

**Figure S1**

*Comparison of MRS trait estimates of those who do not use MRS (ERS only and 0RS classes) and ERS trait estimates of those who do not use ERS (MRS only and 0RS classes) between MM-IRTree and 2RS IRTree.*


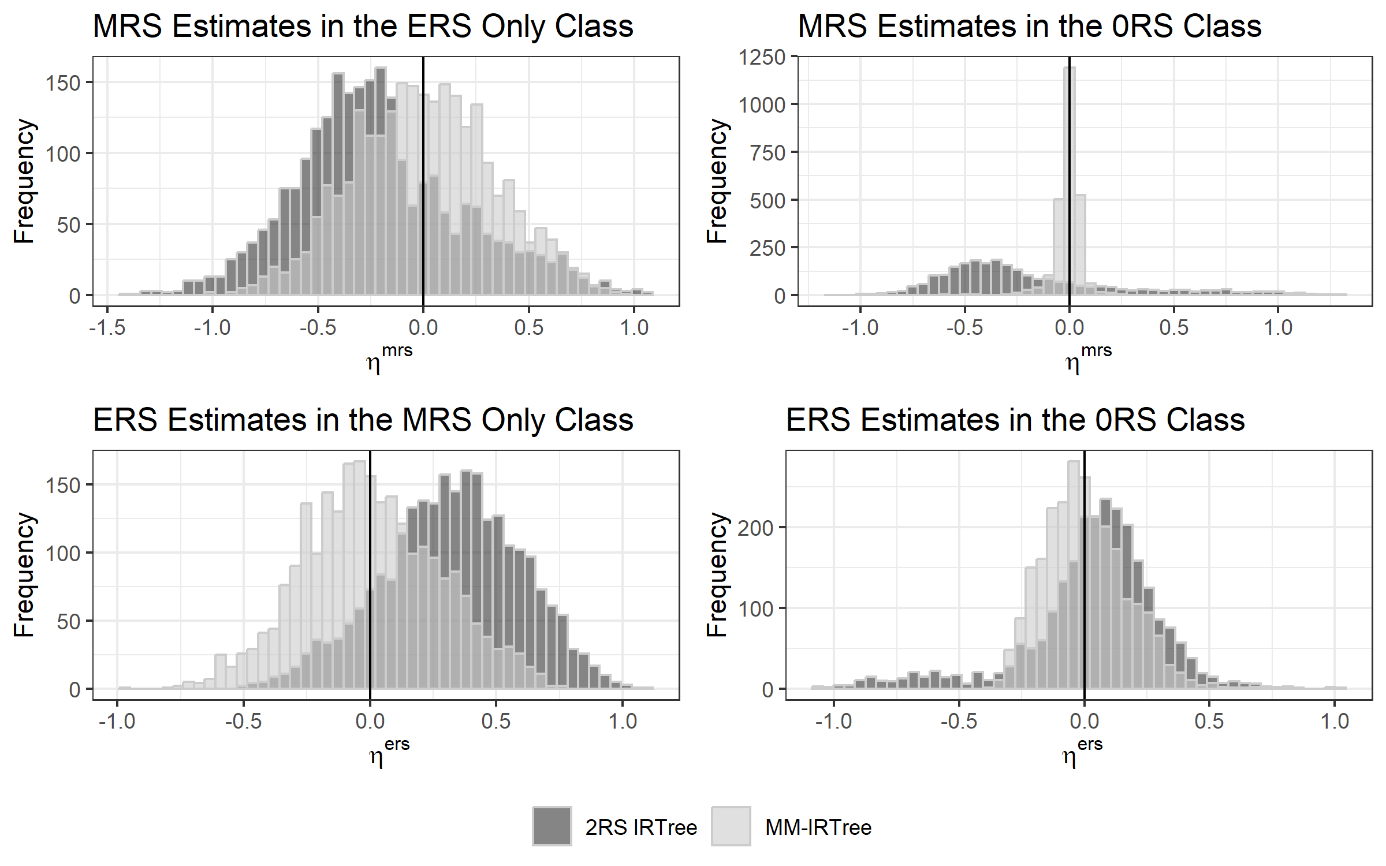


**Figure S2**

*Estimated factor loadings of RS and the substantive trait for different classes. The mean of each univariate posterior distribution is taken as the estimate.*


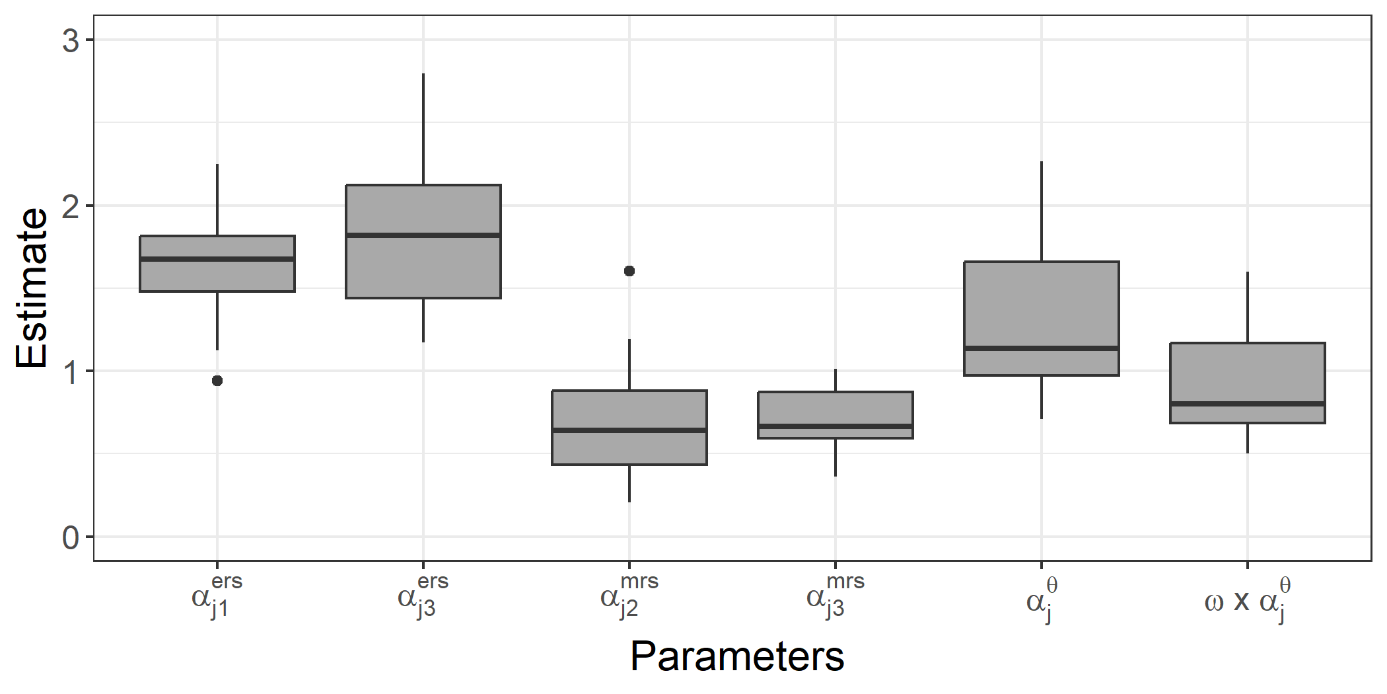


*Note.* $\alpha_{j1}^{ers}$ and $\alpha_{j3}^{ers}$ are factor loadings of ERS trait in “ERS only” and “2RS” classes, respectively. $\alpha_{j2}^{mrs}$ and $\alpha_{j3}^{mrs}$ are factor loadings of MRS trait in “MRS only” and “2RS” classes, respectively. $\alpha_{j}^{\theta}$ are factor loadings of the substantive trait at the first two nodes, their multiplication with the proportionality constraint $\omega$ are factor loadings at the third node.

**Figure S3**

*Estimated node difficulties for different classes. The mean of each univariate posterior distribution is taken as the estimate.*


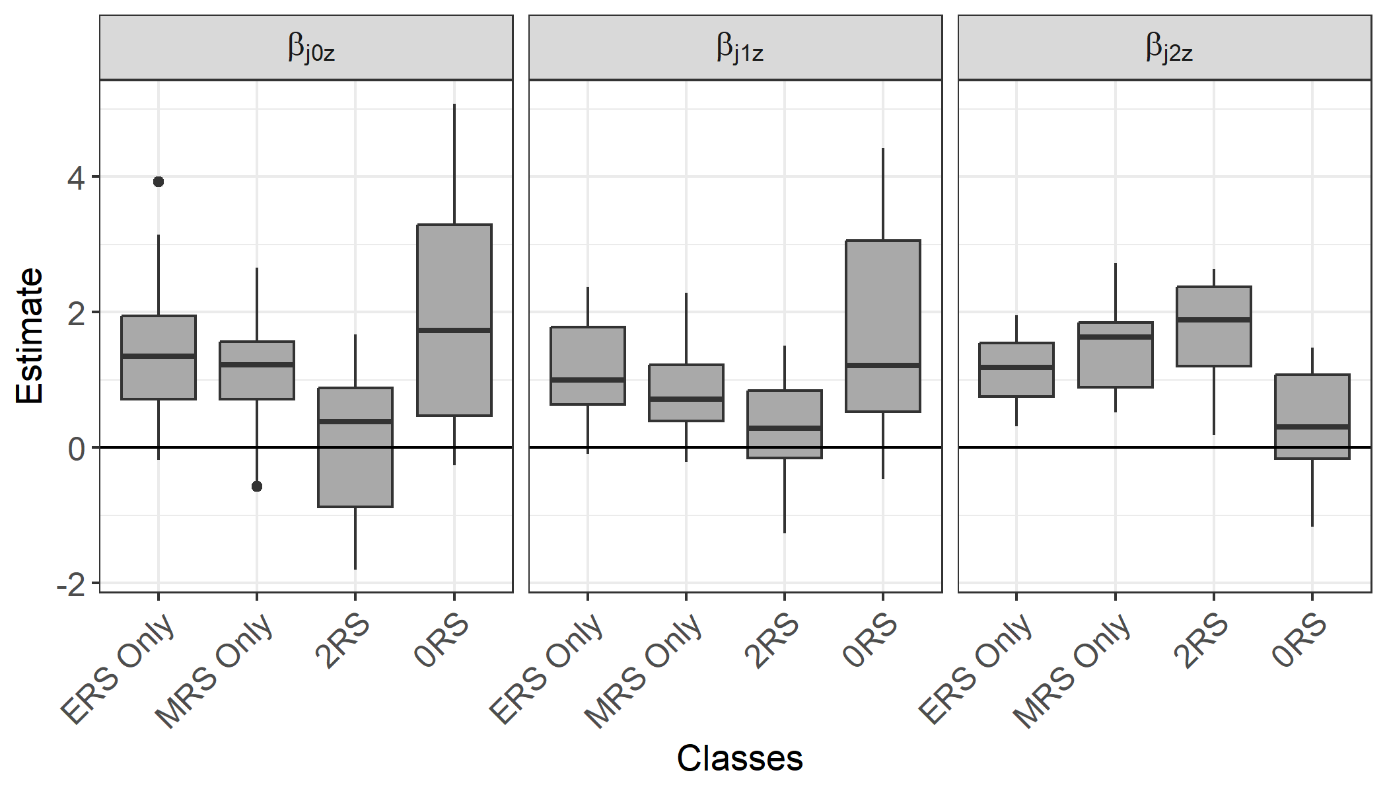


*Note.* $\beta_{j0z}$, $\beta_{j0z}$, $\beta_{j0z}$ are the difficulty parameters of disagreement, neutrality, and extremity nodes, respectively.

**Figure S4**

*Comparison of person sum scores between the observed data set and replicated data sets during MCMC* estimation. Each plot presents the comparison for a different class.
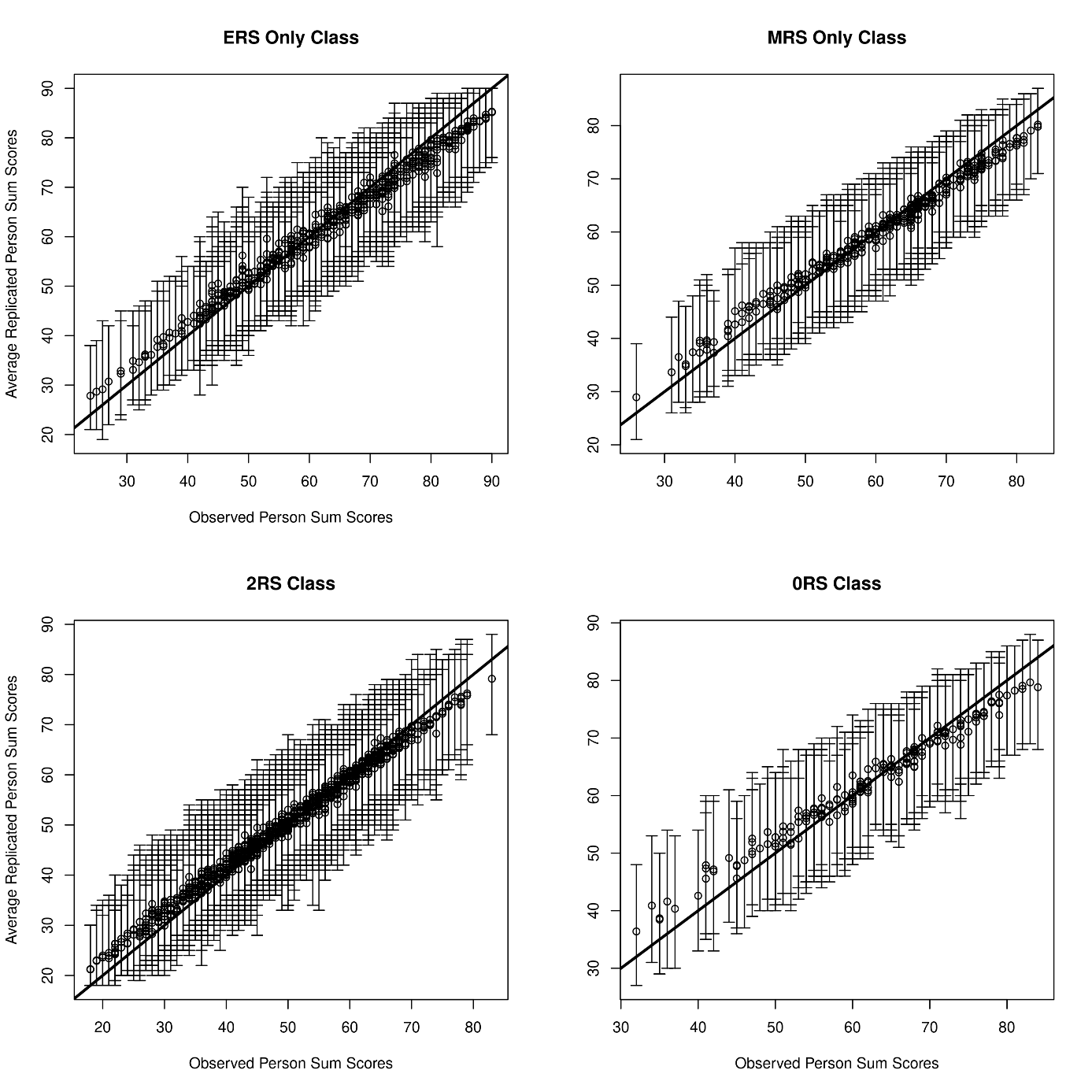


**Figure S5**


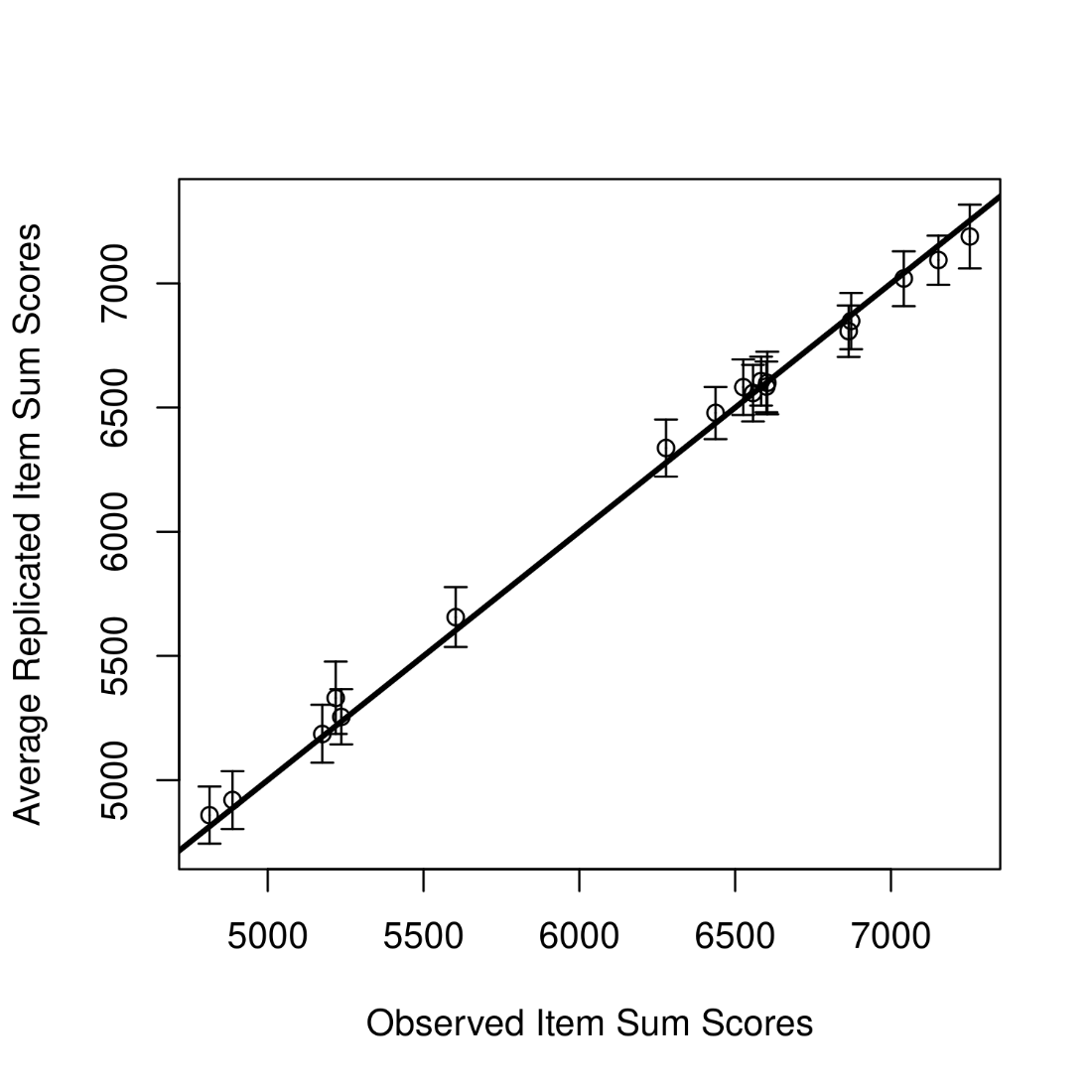
*Comparison of item sum scores between the observed data set and replicated data sets during MCMC* estimation.

**Figure S6**

*Bias and RMSE of the substantive trait* $(\theta)$ *scores for MM-IRTree, ERS IRTree, MRS IRTree, 2RS IRTree, and 0RS IRTree in the “ERS only“ class dominated condition.*


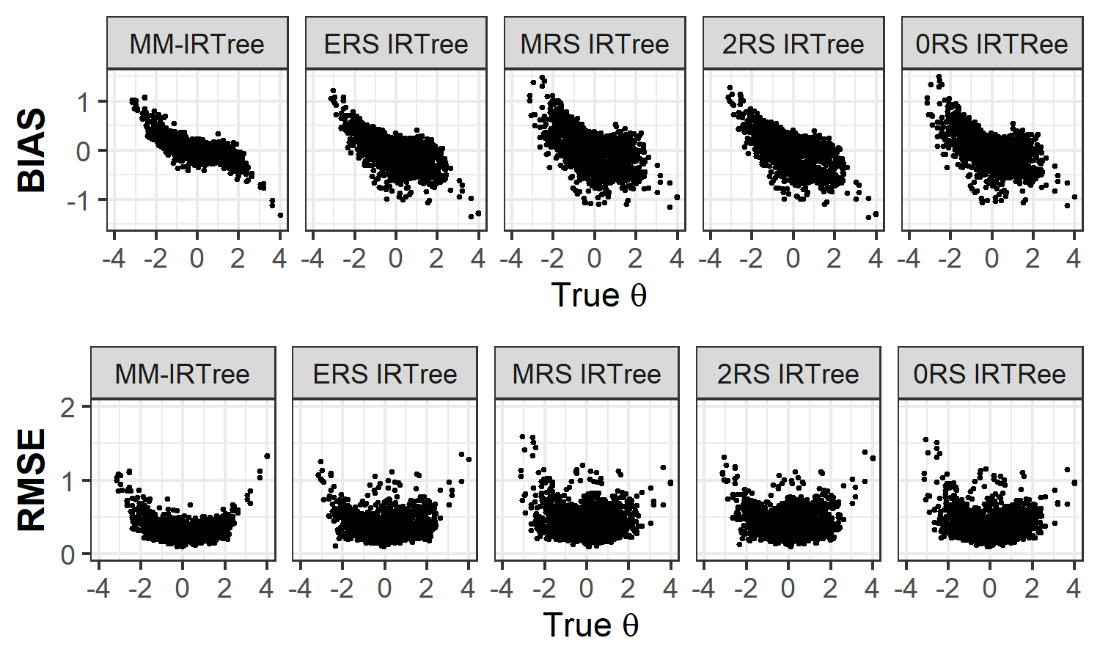


**Figure S7**

*Bias and RMSE of the substantive trait* $(\theta)$ *scores for MM-IRTree, ERS IRTree, MRS IRTree, 2RS IRTree, and 0RS IRTree in the “MRS only“ class dominated condition.*


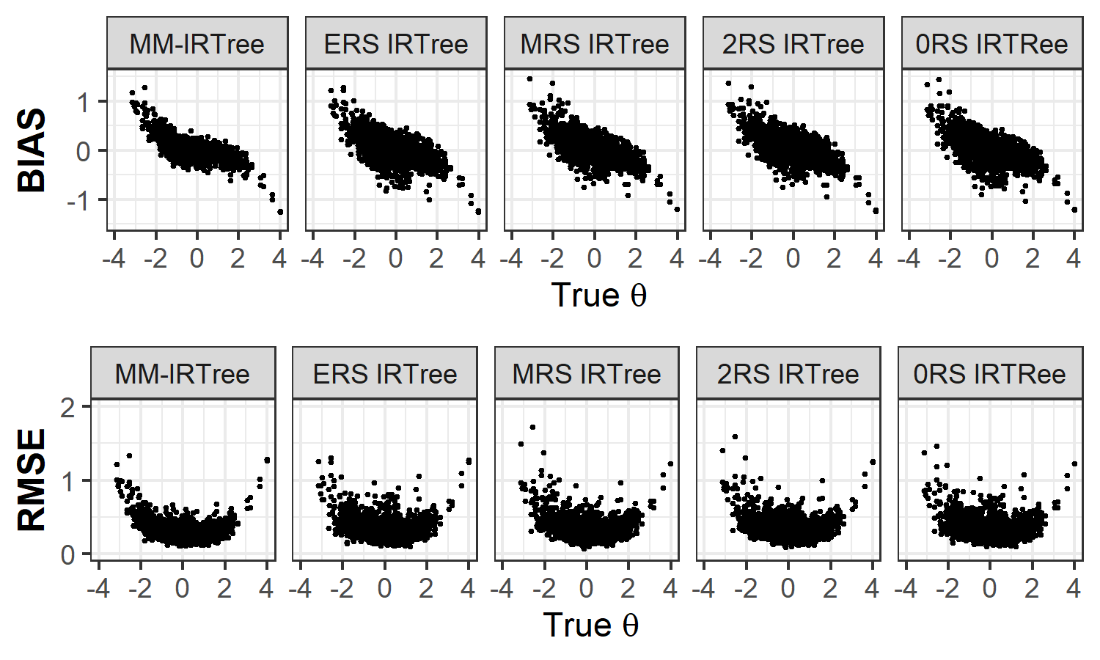


**Figure S8**

*Bias and RMSE of the substantive trait* $(\theta)$ *scores for MM-IRTree, ERS IRTree, MRS IRTree, 2RS IRTree, and 0RS IRTree in the “2RS“ class dominated condition.*


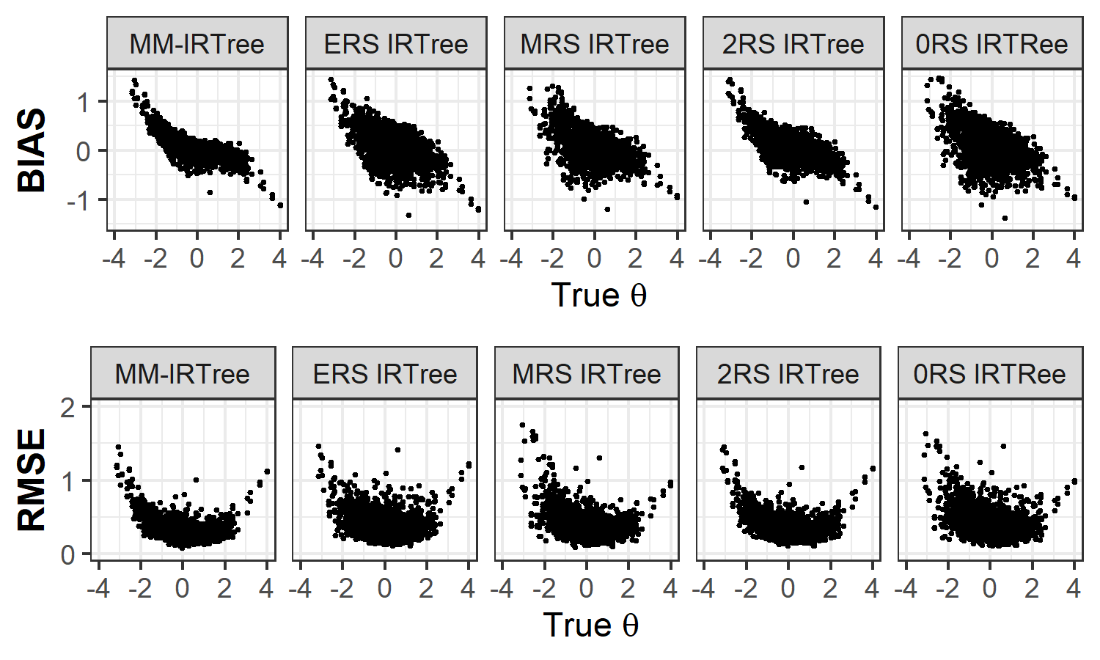


**Figure S9**

*Bias and RMSE of the substantive trait* $(\theta)$ *scores for MM-IRTree, ERS IRTree, MRS IRTree, 2RS IRTree, and 0RS IRTree in the “0RS“ class dominated condition.*


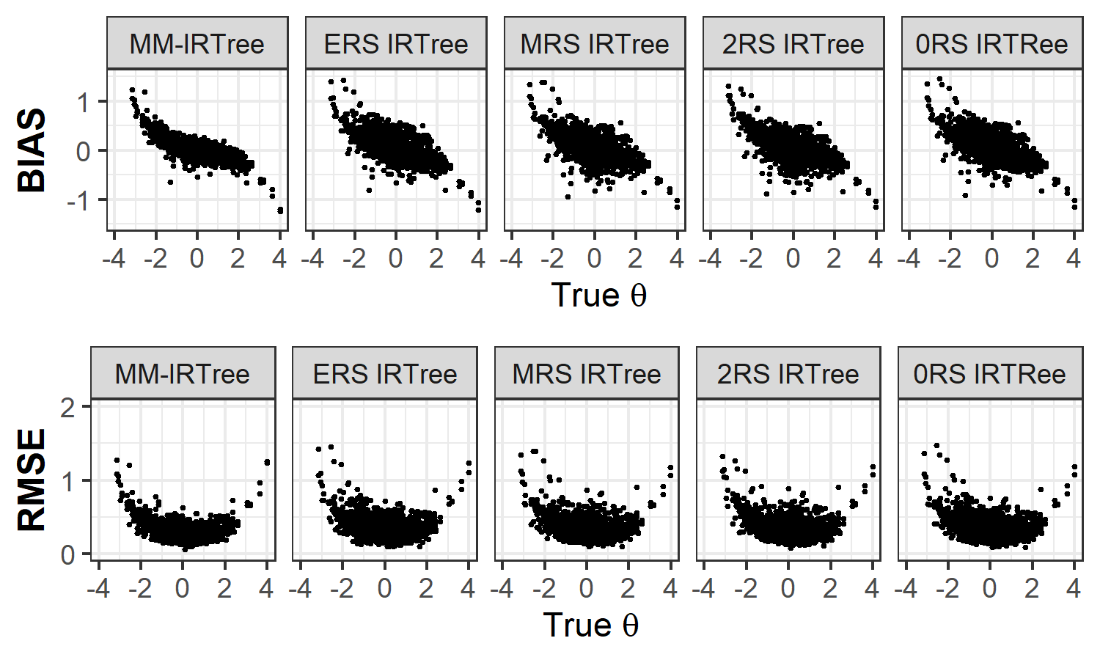


**Figure S10**

*Bias and RMSE of the substantive trait* $(\theta)$ *scores for MM-IRTree, ERS IRTree, MRS IRTree, 2RS IRTree, and 0RS IRTree in the single “ERS only“ class condition.*


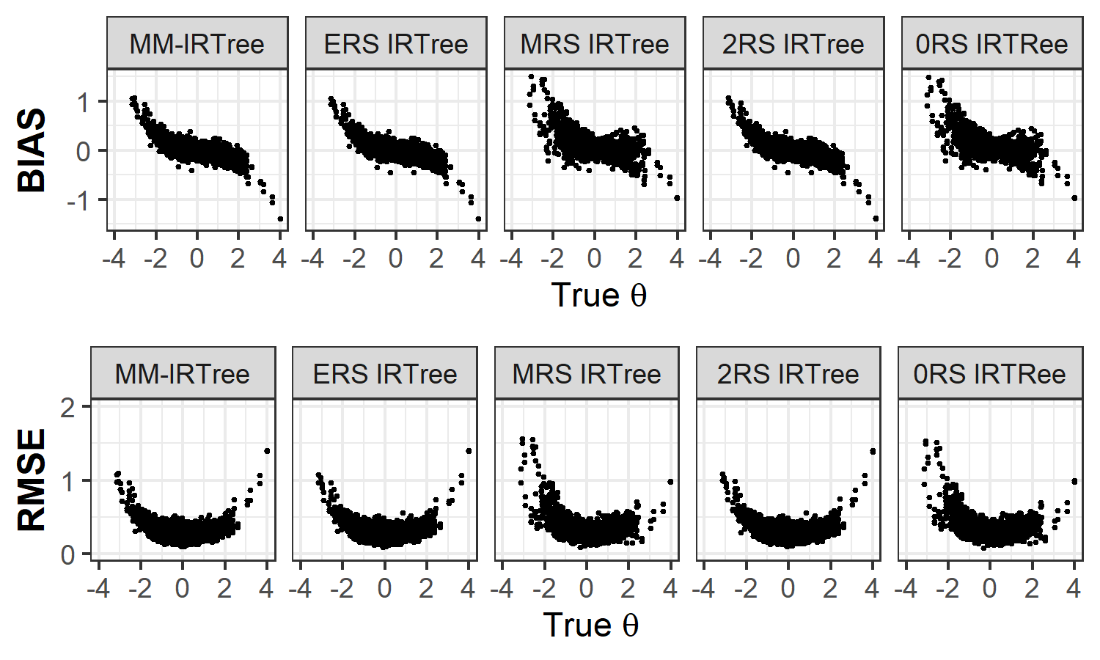


**Figure S11**

*Bias and RMSE of the substantive trait* $(\theta)$ *scores for MM-IRTree, ERS IRTree, MRS IRTree, 2RS IRTree, and 0RS IRTree in the single “MRS only“ class condition.*


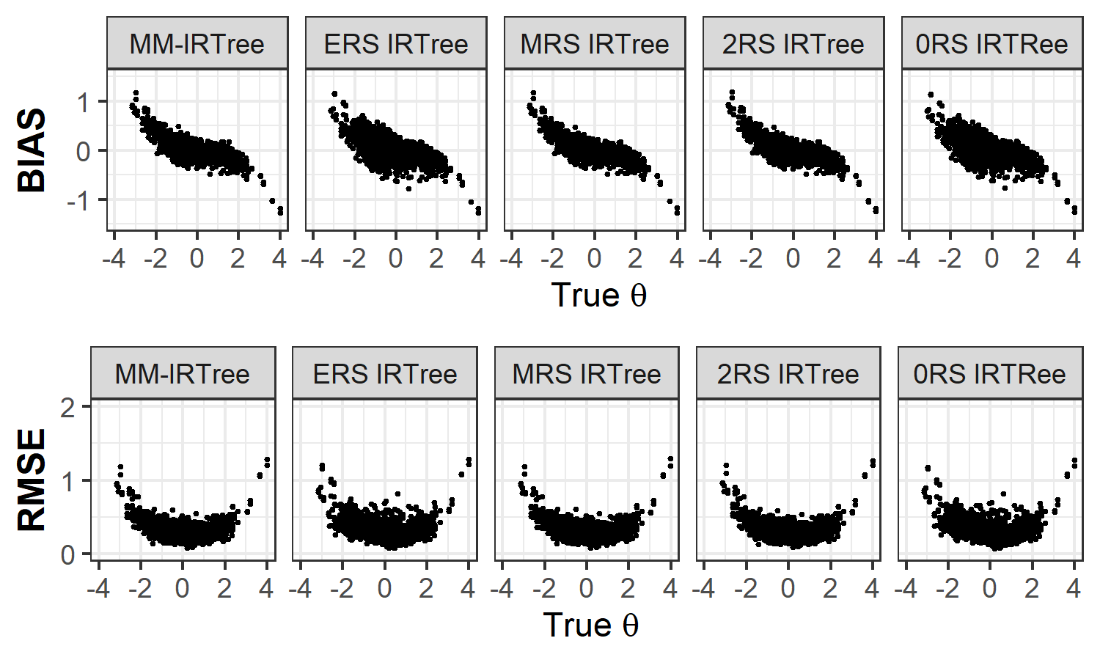


**Figure S12**

*Bias and RMSE of the substantive trait* $(\theta)$ *scores for MM-IRTree, ERS IRTree, MRS IRTree, 2RS IRTree, and 0RS IRTree in the single “2RS“ class condition.*


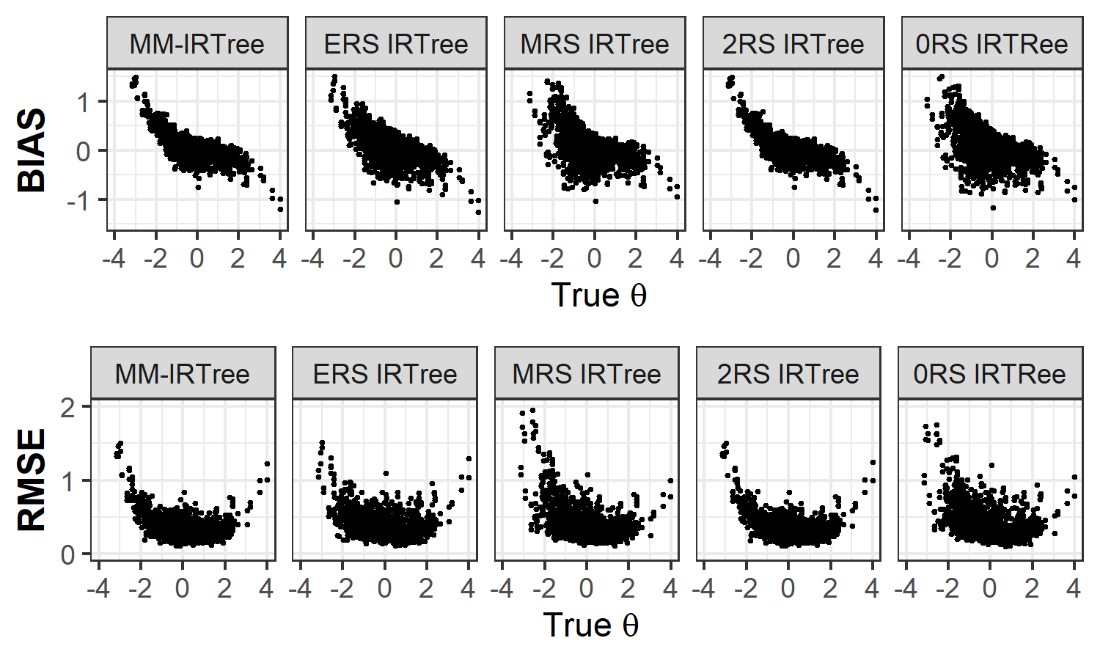


**Figure S13**

*Bias and RMSE of the substantive trait* $(\theta)$ *scores for MM-IRTree, ERS IRTree, MRS IRTree, 2RS IRTree, and 0RS IRTree in the single “0RS“ class condition.*


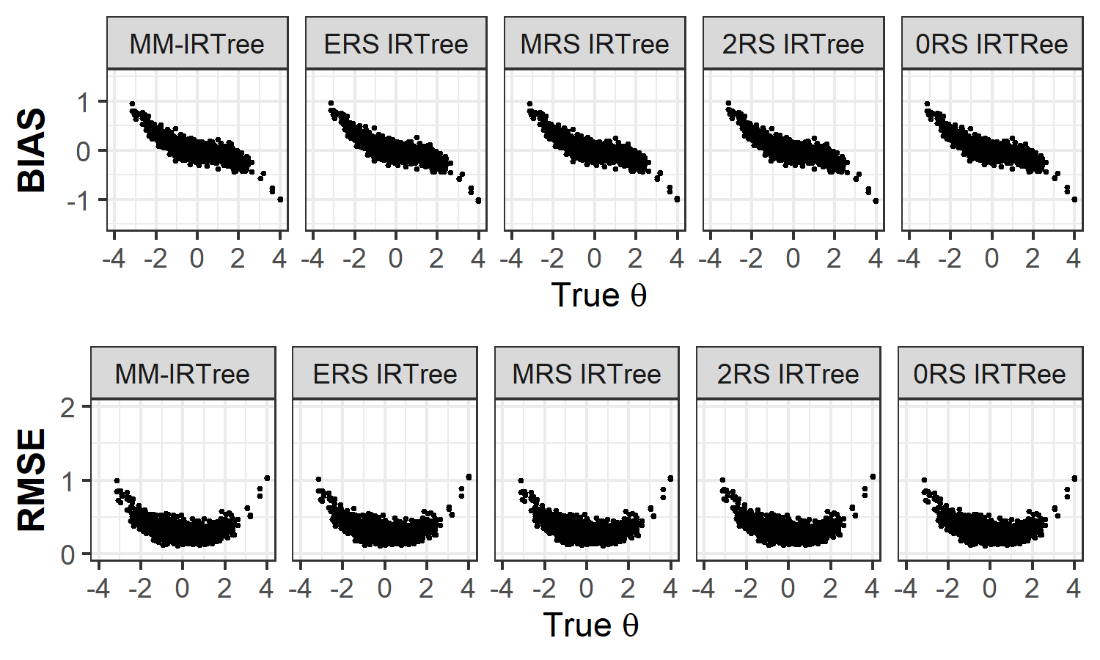


**Figure S14**

*Bias and RMSE of MRS trait scores* $\left( \eta^{ers} \right)$ *obtained with MM-IRTree (left), ERS IRTree (middle), and 2RS IRTree (right) models in the 2RS dominated class proportions condition.*


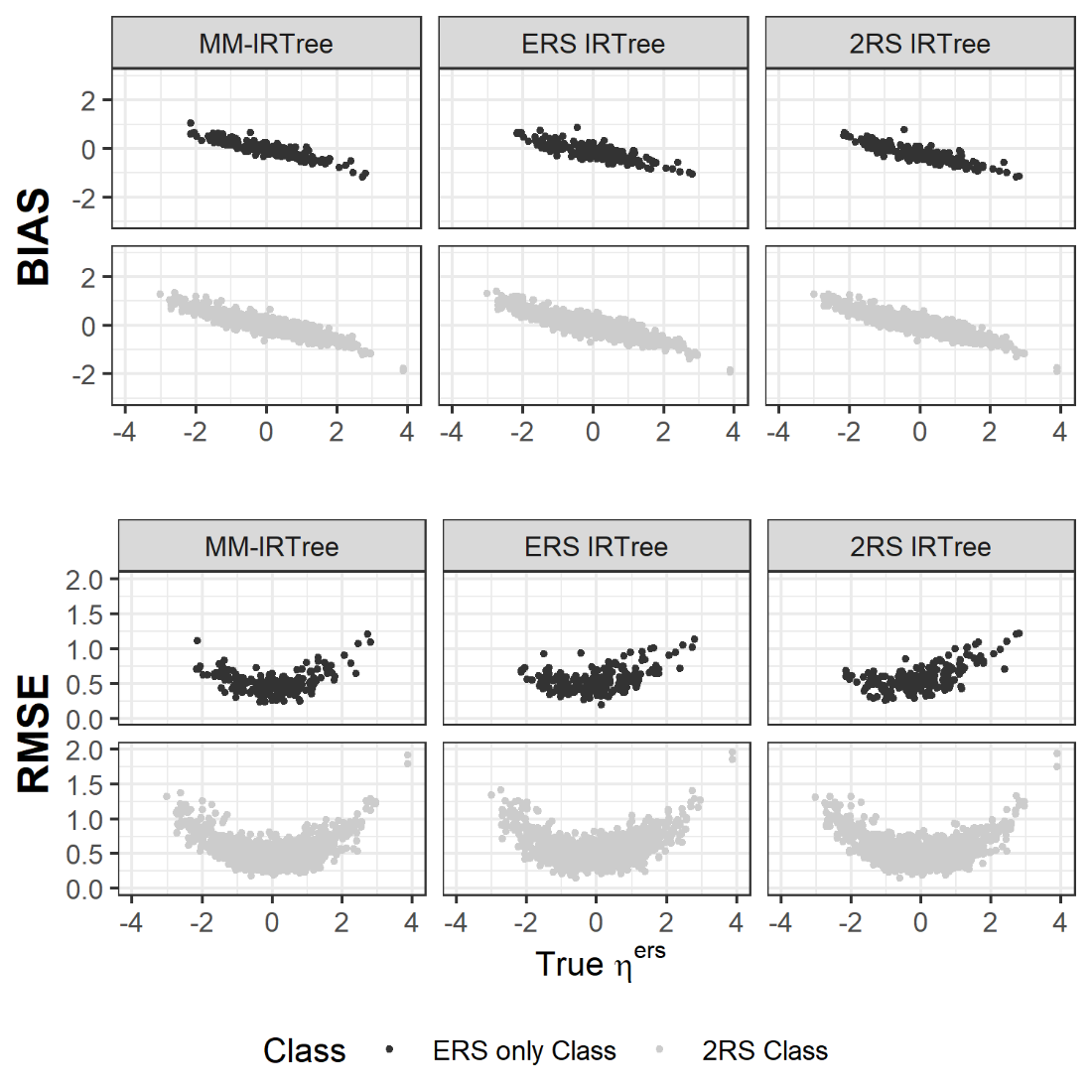


**Figure S15**

*Bias and RMSE of MRS trait scores* $\left( \eta^{mrs} \right)$ *obtained with MM-IRTree (left), MRS IRTree (middle), and 2RS IRTree (right) models in the 2RS dominated class proportions condition.*


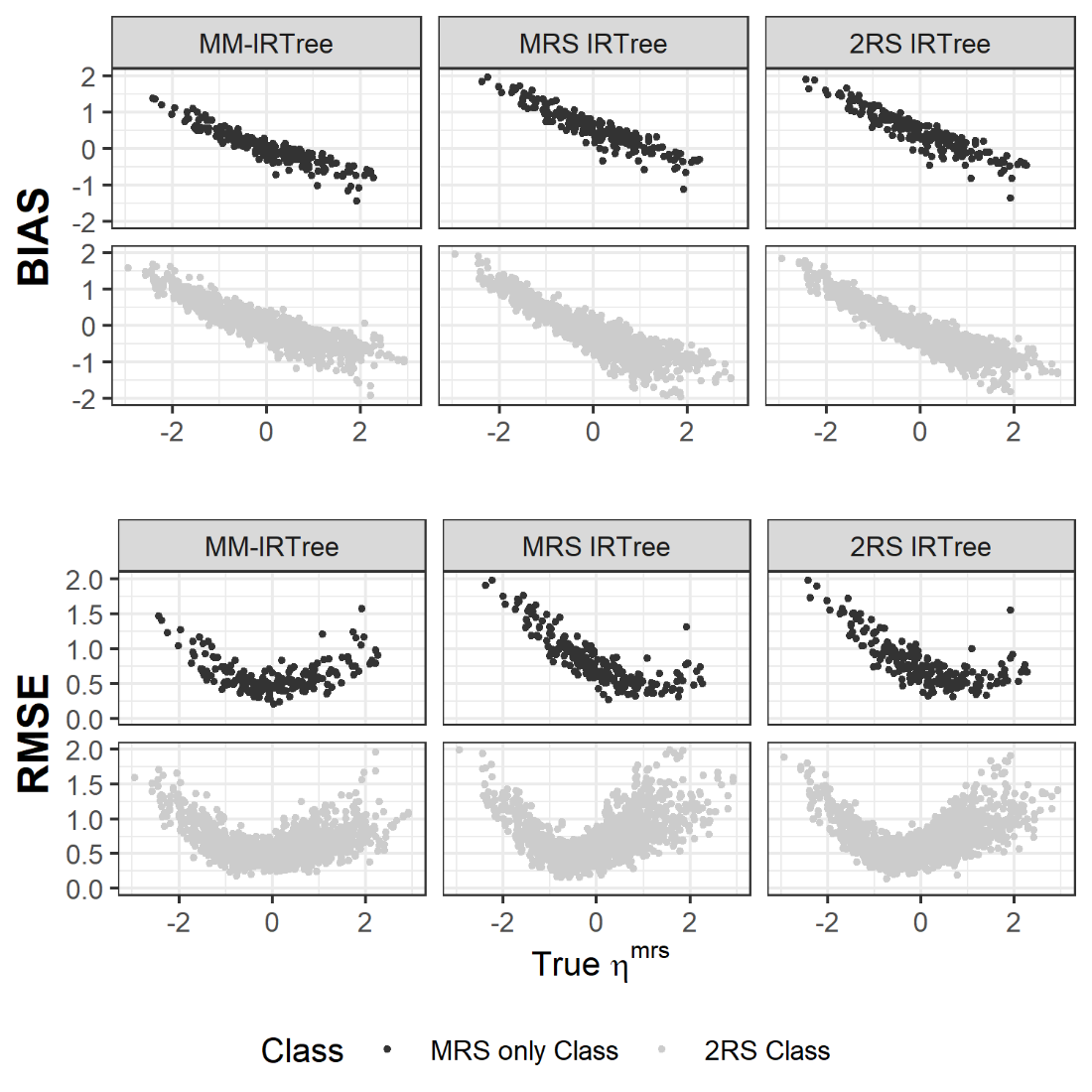

Supplement: sj-docx-1-epm-10.1177_00131644231206765 – Supplemental material for Investigating Heterogeneity in Response Strategies: A Mixture Multidimensional IRTree Approach [file sj-docx-1-epm-10.1177_00131644231206765.docx]
